# Computational Inference of Neural Information Flow Networks

**DOI:** 10.1371/journal.pcbi.0020161

**Published:** 2006-11-24

**Authors:** V. Anne Smith, Jing Yu, Tom V Smulders, Alexander J Hartemink, Erich D Jarvis

**Affiliations:** 1 Department of Neurobiology, Duke University Medical Center, Durham, North Carolina, United States of America; 2 Department of Electrical and Computer Engineering, Duke University, Durham, North Carolina, United States of America; 3 School of Biology and Psychology, University of Newcastle upon Tyne, Newcastle upon Tyne, United Kingdom; 4 Department of Computer Science, Duke University, Durham, North Carolina, United States of America; University College London, United Kingdom

## Abstract

Determining how information flows along anatomical brain pathways is a fundamental requirement for understanding how animals perceive their environments, learn, and behave. Attempts to reveal such neural information flow have been made using linear computational methods, but neural interactions are known to be nonlinear. Here, we demonstrate that a dynamic Bayesian network (DBN) inference algorithm we originally developed to infer nonlinear transcriptional regulatory networks from gene expression data collected with microarrays is also successful at inferring nonlinear neural information flow networks from electrophysiology data collected with microelectrode arrays. The inferred networks we recover from the songbird auditory pathway are correctly restricted to a subset of known anatomical paths, are consistent with timing of the system, and reveal both the importance of reciprocal feedback in auditory processing and greater information flow to higher-order auditory areas when birds hear natural as opposed to synthetic sounds. A linear method applied to the same data incorrectly produces networks with information flow to non-neural tissue and over paths known not to exist. To our knowledge, this study represents the first biologically validated demonstration of an algorithm to successfully infer neural information flow networks.

## Synopsis

One of the challenges in the area of brain research is to decipher networks describing the flow of information among communicating neurons in the form of electrophysiological signals. These networks are thought to be responsible for perceiving and learning about the environment, as well as producing behavior. Monitoring these networks is limited by the number of electrodes that can be placed in the brain of an awake animal, while inferring and reasoning about these networks is limited by the availability of appropriate computational tools. Here, Smith and Yu and colleagues begin to address these issues by implanting microelectrode arrays in the auditory pathway of freely moving songbirds and by analyzing the data using new computational tools they have designed for deciphering networks. The authors find that a dynamic Bayesian network algorithm they developed to decipher gene regulatory networks from gene expression data effectively infers putative information flow networks in the brain from microelectrode array data. The networks they infer conform to known anatomy and other biological properties of the auditory system and offer new insight into how the auditory system processes natural and synthetic sound. The authors believe that their results represent the first validated study of the inference of information flow networks in the brain.

## Introduction

Network flow is distinct from network connectivity. Network connectivity describes the static architecture of a network or, more colloquially, its “wiring diagram.” In contrast, network flow describes the dynamic utilization of a network as a transportation or communication medium. Road maps, electrical grids, Internet backbones, CPU architectures, and anatomical connections in the brain all relate to network connectivity; we call these static structures *connectivity networks*. But traffic congestion, power blackouts, packet routing, computation, and animal behavior are all examples of the phenomena that arise from dynamic flows over these respective connectivity networks; we call these dynamic structures *flow networks*. Although flow networks are constrained to paths present in the respective connectivity networks, knowing network connectivity alone is not sufficient to understand how a network is utilized under different conditions. For example, while a road map shows the physical road networks, it does not have information about which roads are heavily traveled, and when. Similarly, while neural connectivity networks describe the existence of anatomical connections between different brain regions, they lack information about which of those paths are utilized during processing or learning tasks undertaken by the brain. To understand these phenomena, we need flow networks.

Flow networks can be determined by dynamic observation of a system. In the case of determining a flow network describing traffic, this can be done directly by observing automobiles traveling along roads from a traffic helicopter. In the case of the brain, such direct observation is considerably more difficult, requiring monitoring of action potentials traveling along axons. However, we can collect simultaneous recordings of activity in brain regions between which information is flowing. This is analogous in the case of traffic to counting the number of automobiles passing through various intersections. From such observation of variables over time, it is theoretically possible to infer flow networks, or what we call “neural information flow networks” for the brain, which broadly represent the set of paths along which combinations of neural signals (action potentials, end plate potentials, neurotransmitter release, etc.) are transmitted between brain regions during specific processing tasks. In relation to definitions commonly used within the neuroimaging field [1,2] but with historical precedents in electrophysiology [3,4]—where functional connectivity refers to statistical patterns with no causal implications and effective connectivity refers to causal neural interactions defined as transmitted neural signals—we consider a neural information flow network to be representing effective connectivity; this is distinct from the anatomical connectivity network.

Attempts have recently been made to infer neural information flow networks from simultaneous recordings of electrophysiology activity in multiple brain regions using microelectrode arrays [5,6]. Depending on how the data are processed and where the electrodes are placed, the putative neural information flow networks that have been inferred represent the transmission of information between either individual neurons or populations of neurons, in either the same or different brain regions. Existing methods are commonly based on simple cross-correlation or coherence analyses between pairs of electrodes [5–7], but such methods cannot resolve direct from indirect flow (flow via one or more *measured* intermediary regions), leading to highly interconnected networks [8]. Attempts have been made to limit network recovery to only direct information flow by analyzing more than two electrodes at a time [8–13]. However, most of these methods assume linear relationships between measured neural activities, when such relationships are known to be nonlinear [8,9] (but see [5] for use of piece-wise linear methods to approximate nonlinearity). This mismatch of statistical assumption with biological reality is a potential source of inaccurate inference. Moreover, none of the inferred neural information flow networks generated by any method that we are aware of has been validated against known anatomy, despite the fact that in certain neural systems, networks of anatomical connectivity are well-characterized, providing knowledge of the paths over which information must flow.

Recently, we [14–17] and others [18] developed, improved, and demonstrated the ability of dynamic Bayesian network (DBN) inference algorithms to infer transcriptional regulatory networks from gene expression microarray data collected from simulated gene networks. These inference algorithms have also been applied to experimental gene expression data [17,19,20], but the true topologies of transcriptional regulatory networks are generally not known, so validation is more challenging than with simulated networks (but see [21] for a recent successful effort to recover a well-studied signal transduction pathway). Here, we tested and validated whether the DBN inference algorithm we developed for inferring transcriptional regulatory networks from gene expression microarray data would be successful at inferring information flow networks from microelectrode array data collected from a neural system of known connectivity—the songbird auditory pathway [22]. This seemed plausible because in order to accurately learn complex network architectures, inference algorithms can require large numbers of observations for each variable (e.g., thousands of time points) [16], which is difficult and expensive to collect with microarrays but not with microelectrode arrays. We further reasoned that DBN inference algorithms might be particularly effective for two reasons. First, Bayesian networks can model multifactor, combinatorial, and stochastic interactions that are either linear or nonlinear [23], providing an appropriate statistical framework for the neural system. Second, Bayesian networks model only direct statistical dependencies among included variables, whether these variables are observed (i.e., measured) or hidden—that is, if two variables statistically interact only indirectly through a third variable, Bayesian networks model only the two direct statistical interactions [23]. In particular, when using a Bayesian network inference algorithm to infer relationships among only a set of measured variables, two indirectly interacting variables will not be connected if the third intervening variable is also measured. If the third variable is not measured, it is possible to find a statistical relationship between the first two; however, DBNs with appropriately chosen sampling intervals can be used to minimize such unintended indirect relationships [15,16]. We found that our DBN inference algorithm, without modification, successfully infers neural information flow networks that match known anatomy from electrophysiology data collected from the auditory pathway of awake, freely moving songbirds. In contrast, we found that a commonly used linear method, never before validated against known anatomy, had many errors.

## Results

### Inference of Neural Information Flow Networks

**Figure 1:**
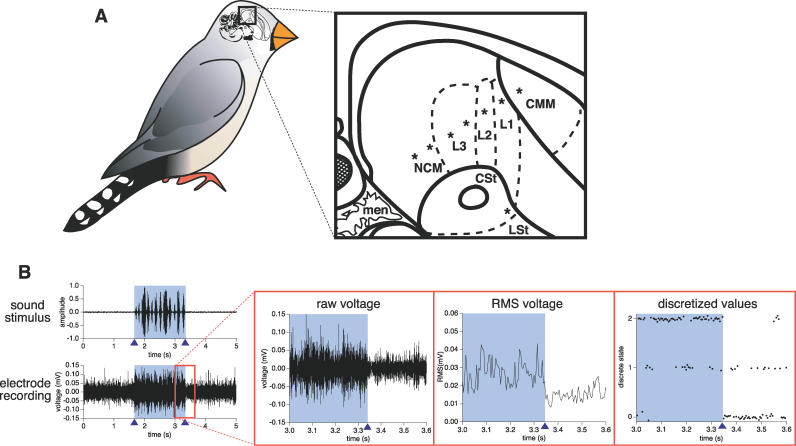
Electrophysiological Recording from Songbird Auditory Forebrain (A) Electrode placements. Zebra finch drawing shows a sagittal brain section (to scale, ∼1.1 cm in length); the boxed area highlights the auditory regions, magnified on the right. Microelectrode arrays were placed in a linear posterior–anterior orientation (asterisks [*] indicate electrode locations), in nearly all known major auditory pallial (nidopallium caudale mediale [NCM], fields L3, L2, L1, and caudal medial mesopallium [CMM]) and striatal (caudal striatum [CSt]) regions. Of the 48 electrodes placed in the six birds, two ended up outside of the auditory pathway (one in the lateral striatum [LSt] and one in the meninges [men]). The anatomical terms used are those of the new avian brain nomenclature [56]. Solid lines, brain subdivisions; dashed lines, auditory regions. (B) Data processing. From left to right: amplitude envelope of a song stimulus above measured voltage changes sampled from an L2 electrode during stimulus presentation; magnification of the voltage changes; RMS values of these voltages; three-state discretization of these RMS values (presented with jitter for clarity). Shaded region, sound; triangles, onset and offset of sound.

We developed linear arrays of eight fluorescently labeled microelectrodes for recording multi-unit neural activity in the brains of awake, freely moving songbirds (for details, see Methods, Protocol S1, and Figure S1). These arrays were placed in cerebral auditory regions (NCM, L3, L2, L1, CMM, and CSt; Figure 1A) of six female zebra finches, and voltage changes in the populations of neurons surrounding the electrodes were recorded while the birds were presented with four digitized sound stimuli (Figure 1B) on each of four days (only three days for bird 1). These stimuli were 20 repetitions of two natural sounds (two of eight different zebra finch songs for each day) and two synthetic sounds (white noise and amplitude-modulated white noise patterned on one of the zebra finch songs of that day); natural and synthetic sounds are known to induce different firing responses in the songbird auditory pathway [24,25]. To estimate the collective electrical activity of the population of neurons surrounding an electrode, a root mean square (RMS) transformation of the recorded multi-unit neuronal voltages was computed in 5-ms time intervals (Figure 1B); this time interval was chosen because it takes an estimated 5–10 ms for a neural signal to travel through one synaptic connection [26], and simulation studies with our DBN algorithm revealed that there is an optimal sampling rate for the most accurate inference possible, and that this sampling rate should be equal to or slightly smaller than the time required for a variable to affect a directly adjacent variable in the network [15,16]. Discretization permits us to learn arbitrary combinatoric (i.e., any form of linear or nonlinear) relationships between measured activities at the electrodes, and so the RMS voltage data from the 20 repetitions of a stimulus (∼20,000 data points on average) on each day were discretized with a quantile-based method into three states (Figure 1B) and then provided to our DBN inference algorithm. This yielded a total of 16 networks for each bird (four stimuli on each of four days; 12 networks for bird 1). These networks represent the simplest models for suitably explaining the statistical relationships among the measured activities of neuron populations near each electrode over time.

**Figure 2:**
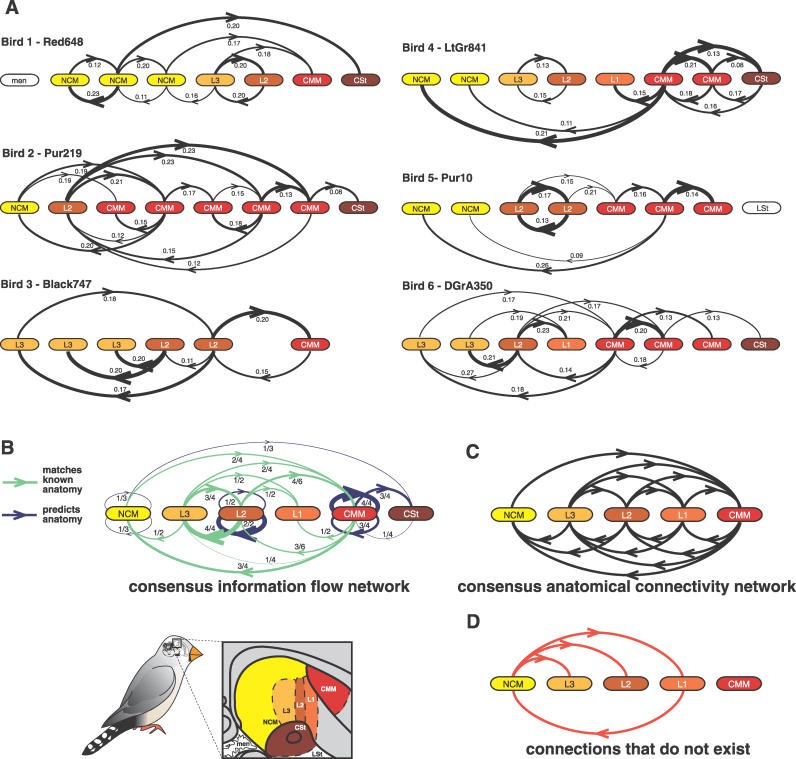
Generation of Information Flow Networks from Songbird Brain Using a DBN Algorithm (A) Inferred neural information flow networks. Networks show significant interactions compiled across the 16 (or 12 for bird 1) inferred networks from hearing all stimuli across all days. Line thickness is proportional to the square of link occurrence frequency; numbers denote average influence scores. The order of the variables in the recovered DBN is the order of electrodes in the brain from posterior (left) to anterior (right). Multiple electrodes were sometimes within the same region. Brain regions are color-coded to highlight differences in electrode placement across birds. Bird 3 had two electrodes that were short-circuited and transmitted no signal; thus, these are not shown. (B) Consensus flow network compiled from the interactions of all birds from (A). Each oval represents one region. Lines connecting a region to itself represent those between two or more electrodes in the same region; those directed to the right indicate an interaction from one electrode to another anterior to it in the same region; those to the left indicate the reverse. Green lines, known anatomical paths; blue lines, predictions about anatomical connections between regions where connectivity is currently unknown. It was not possible to recover internal interactions in L1 or CSt, as no bird had more than one electrode in these regions. Fractions represent the number of birds in which such an interaction occurred out of the number of birds in which such an interaction was possible. Line thickness is proportional to the square of these fractions. (C) Consensus connectivity network of known anatomical connections of auditory forebrain regions determined across many birds from multiple studies [22,25,57–59]. Connectivity of CSt is not well-characterized and therefore not shown. (D) The four anatomical connections among auditory regions known not to exist.

When supplied with the discretized RMS electrophysiology data, the algorithm successfully produced networks with interactions that were significantly consistent for each of the six birds (Figure 2; *p* < 0.02, Monte Carlo analysis). The term *interaction* refers to a significantly reoccurring link across the 16 networks per bird (12 for bird 1), while the term *link* refers to a directed relationship between two electrodes found in an individual network—i.e., putative transmission of neural signals between the measured neuronal populations. The majority of links in the inferred networks (65%–83%, depending upon the bird) contributed to these significantly consistent interactions. No two birds had identical networks, which was expected because no two birds had identical electrode placements (within or across brain regions) due to the variable nature of microsurgery (Figure 2A). The differences in interactions among birds and among electrodes within the same brain region in individual birds may be explained by the known medial–lateral topography in connectivity of the songbird auditory system [22]; interactions between electrodes which happened to be in the same medial–lateral plane would be more likely to interact. This appeared to be the case, as birds with similar electrode placements appeared to have more similar networks; for example, birds 4 and 5 had the closest similarity of electrode placements across brain regions and within the medial-to-lateral plane (Figure S1C), and their networks appeared to be more similar to each other than to other birds' (Figure 2A). Another possibility is that different networks are due to true physical anatomical connectivity or connection density differences across birds, although this remains to be tested.

To rule out the possibility that the DBN algorithm would infer links simply by chance, we supplied it with three types of randomized data: 1) the real RMS values collected from each electrode but shuffled randomly to destroy possible dependencies, 2) simulated random values with the same range as the real RMS values but generated independently for each electrode to preclude possible dependencies, and 3) simulated random values with the same range and same first-order temporal dependencies as the real RMS values but generated independently for each electrode. In all three cases, no links were ever inferred by our DBN inference algorithm, suggesting that the links inferred from the real electrophysiology data are not the product of chance.

To determine whether the DBN algorithm would *not* infer links between variables known to be conditionally independent, we passed it electrodes from all six birds simultaneously (46 electrodes at once), without providing the algorithm with information about which electrodes came from which birds. Since the birds' auditory systems respond to the sound stimuli, we added one more variable: a binary indicator of the presence of sound versus silence. Since this variable forms the only commonality between birds, we know that individual birds' electrodes therefore are conditionally independent given this sound variable. The algorithm correctly inferred conditional independence across birds: no interactions were found between electrodes of different birds (Figure S2). As expected, there were interactions from the sound variable to the electrodes, but none in the reverse direction, showing correct direction of causation. Interestingly, interactions were found from sound to more than just the main auditory input, L2. Thus there is variability related to sound in the electrode recordings that cannot be explained by interconnections among them, indicating that we have not sampled the entire system in any one bird; however, this is a fact we already knew. Encouragingly, 81 ± 5% (mean ± SE) of the interactions found within the birds were identical to those found when analyzing birds individually, indicating that the addition of a non-neural variable (sound) did not greatly change the dependency structure.

**Figure 3:**
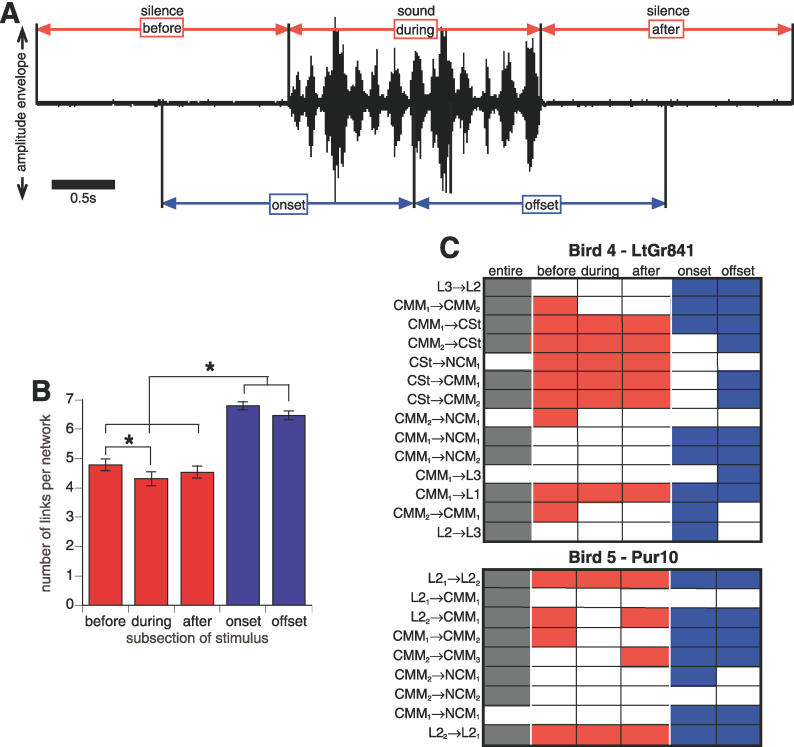
Analysis of Networks Generated from Birds Hearing Stimulus Subsections (A) Sample amplitude envelope of a 6-s song stimulus, showing equal size stimulus subsections. Scale bar = 0.5 s. (B) Average number of links per network for each subsection. Asterisks (*) indicate Bonferroni-corrected significance at α = 0.05 (Table S3). Error bars represent standard errors of the mean. (C) Significantly consistent interactions recovered from subsections compared with those recovered from the entire stimulus period, for two example birds. Datasets are shown in columns, interactions in rows. Filled cell indicates that the interaction was significantly consistent (grey, entire stimulus; red, nontransition subsections; blue, transition subsections). Arrows (→) indicate direction of flow. For multiple electrodes within the same brain region, higher subscript number indicates more anterior electrode.

To test whether there were specific portions of the data that were most informative for DBN network inference, we supplied the algorithm with subsets of the data from five subsections of the stimulus period: before, during, or after the stimulus, and equally sized subsections covering the onset and offset transitions of sound stimuli (Figure 3A; this procedure was performed for each bird individually). We found that the DBN algorithm inferred networks from all stimulus subsections (Figure 3A; Figure S3), including the two silent periods. The inferred networks in the silent periods suggest that even in a quiet soundproof room, an animal's auditory system is processing ambient information and that the dynamics of baseline brain activity provides sufficient information to infer network flow. The majority (82 ± 3%) of interactions recovered per bird from each stimulus subsection matched a subset of the interactions recovered from the entire stimulus; the remaining 18 ± 3% could represent real information flow differences specific to the stimulus subsections. While there was no difference across subsections in the number of significant interactions found, more links per network were recovered from the onset and offset transitions (Figure 3B; statistics in Table S1), indicating that the transition subsections either provided more measurable statistical dependencies for the algorithm or that more information is flowing during the transitions. However, the percentage of both interactions and links that matched interactions from the entire stimulus were similar across all subsections, indicating that transition and nontransition subsections provided equivalent information about the topology of the inferred flow network (Figure 3C; Figure S3 and Table S1).

### Validation of Information Flow Networks

We tested whether the inferred links conform to known properties of the auditory pathway. First, all influence scores (range −1 to 1; see Methods) were positive (Figure 2A), consistent with these pallial regions being dominated by excitatory (glutamatergic) rather than inhibitory (GABAergic) synaptic neurotransmitter receptors [27,28]. Second, electrodes accidentally placed outside the auditory pathway (one in the non-neural meninges [men] of bird 1; one in the nonauditory lateral striatum [LSt] of bird 5) were the only electrodes that never linked in any of the networks. Third, shorter links were found disproportionately more often (Figure S4; statistics in Table S2), consistent with the fact that regions in the auditory pathway that are closer to one another have a higher density of anatomical connections between them [22]. Finally, the consensus-inferred neural information flow network generated from networks across all birds (Figure 2B) conformed to a subset of known anatomical connections in the connectivity network (Figure 2C), as desired. Where connections are known to exist, all recovered significantly consistent interactions matched them (Figure 2B, green lines). Where connections are known not to exist—NCM does not project axons either to L3, L2, or L1, nor does L1 project to NCM [22,25]—the DBN algorithm did not recover interactions and in fact significantly avoided the corresponding links in three of the four cases (*p* < 0.001 – *p* = 0.009 for links out of NCM, *p* = 0.3 for L1 to NCM, Monte Carlo analysis; Figure 2D). Additionally, the fact that there were no links at all with meninges and LSt represent another 16 consensus interactions that were significantly avoided by the algorithm (10 for meninges and 6 for LSt; *p* < 0.001 for both, Monte Carlo analysis).

Although all recovered interactions that matched known anatomical paths occurred significantly above chance and no interactions were found along paths known not to exist in the anatomy, these comparisons are made against a background of a high degree of anatomical interconnectivity, possibly making it easy to find matches to known anatomy by chance (which will be an issue with most sensory neural systems). Thus, we performed additional analyses and found that despite the dense connectivity, it is actually harder than one may intuit to pass this test. In the consensus network, with 16 possible valid paths and four possible invalid paths, the probability of choosing 12 interactions which are all valid by chance is *p* = 0.014 (hypergeometric test). A more robust analysis on the actual nodes and interactions within auditory regions of each bird, where we have 151 possible valid paths and 19 possible invalid paths, revealed that the probability of choosing 38 valid interactions as the algorithm did is *p* = 0.005. When we include the nonauditory regions, there are then 47 possible places in which to place an interaction that is known to be false. The DBN algorithm did not place interactions in any of these 47 places, with the probability of this occurring by chance being *p* = 9.2 × 10^−6^.

The anatomical connectivity of CSt is not well known so we could not evaluate the accuracy of recovered interactions between CSt and other auditory regions (Figure 2B, blue lines). These links represent testable predictions about the existence of novel anatomical connections. Preliminary results suggest that CSt does receive connections from at least L1 and CMM [22], and we found statistically significant interactions of CSt mainly with CMM. Since anatomical connections within a single auditory region are not well known, we also could not validate the interactions within NCM, L2, or CMM (Figure 2B, blue lines); however, such interactions are highly plausible given that internal connectivity within brain regions is common.

The inferred flow networks also conformed with measured physiology of the auditory pathway, specifically onset timing of neural firing in response to a stimulus. After presentation of an auditory stimulus to a songbird, the primary auditory region L2 has on average the quickest onset (∼10 ms), followed by L3, L1, and CMM approximately simultaneously (∼14–15 ms), and followed finally by NCM and CSt (∼30 ms) [29] (Figure S5). This is consistent with our consensus information flow network (Figure 2B), in which after a neural signal arrives in L2, activity flows to L3, L1, and CMM simultaneously, and then to NCM (from both L3 and CMM) and CSt (from CMM) (Video S1). Although our networks conform to measured onset timing, the DBN infers more complex interactions than is possible to infer from onset timing alone. For example, analysis of onset timing alone would not be sufficient to infer the pattern of flow from L3 and CMM to NCM, or to infer more complex patterns of flow such as reciprocal feedback loops. Feedback loops are important in controlling and stabilizing a multitude of biological systems, and the neural information flow networks we recover contain these loops. We observe them between L2 and L3, among multiple regions with CMM, and within NCM, L2, and CMM in birds for which multiple electrodes were present in those regions (Figure 2B; Video S1). Taken together, the above validation against known anatomy and physiology suggests that the inferred networks reflect real neural information flow along anatomical paths among populations of neurons.

### Comparison with a Linear Inference Algorithm

We wanted to see if the performance achieved by the DBN algorithm might be achieved by other algorithms applied to the same datasets, so we compared it to a state-of-the-art linear inference algorithm: partial directed coherence (PDC) [11]. PDC is an advanced form of a lineage of linear coherence analyses that have been developed for electrophysiological analysis, building upon previous work in Granger causality [30], directed coherence [31], and the directed transfer function [32]; e.g., [8,10,12]. We first applied PDC to the first two types of random data that we supplied to the DBN algorithm (the third type is not applicable to PDC, see Methods). In the case of simulated random values, PDC inferred some spurious links at a low frequency: only two links across all the simulated random datasets. However, in the case of shuffled datasets, while there were only two links inferred across the datasets from five of the birds, the other bird (1) had 41 links. In this bird, a Monte Carlo analysis found four significant interactions (three involving the non-neural meninges). Therefore, unlike the DBN, PDC inferred spurious interactions from randomly shuffled data, indicating a tendency to produce false positives from random data, a behavior also noted by its authors [33].

**Figure 4:**
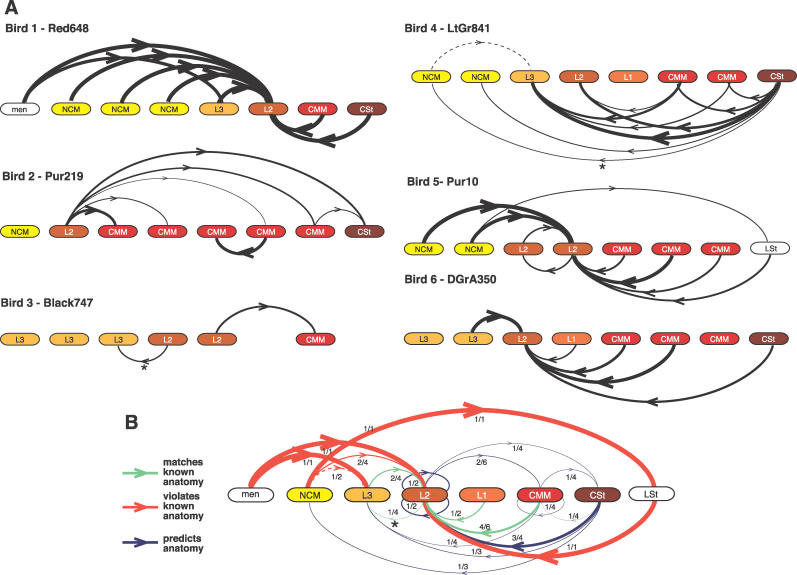
Neural Information Flow Networks Inferred by PDC for Each of the Six Birds (A) and the Resulting Consensus Network (B) Explanation of networks is the same as in Figure 2A and 2B, respectively. Asterisks (*) indicate the two interactions in individual birds (A) and the interaction in the consensus network (B) that drop out if data segments showing a poor model fit are removed from the analyses; dashed line represents the one interaction added when these segments are removed; approximately 10% of the segments for each bird show a poor model fit for PDC. In (B), green lines, known anatomical paths; blue lines, predictions about connections between regions where connectivity is currently unknown; red lines, conflicts with known anatomy.

When we applied PDC to the real data and generated summary networks as before, it produced networks from each of the 16 (12 for bird 1) datasets per bird, and these networks had significantly consistent interactions (Figure 4A). However, when compared with known anatomy, both when using all data and when excluding those segments where PDC diagnostics indicated a poor model fit (see Methods), PDC performed suboptimally (Figure 4B versus Figure 2C). First, the PDC networks contained significantly consistent, but apparently false, interactions between auditory electrodes and both non-neural and nonauditory electrodes (meninges in bird 1; LSt in bird 5), in conflict with known anatomy and brain function. Second, within the auditory pathway electrodes, PDC found significantly consistent, but apparently false, interactions from NCM to L3 and L2, two of the four connections known not to exist, in conflict with known auditory pathway anatomy. Third, the inferred networks produced by PDC tended to be dominated by interactions originating or terminating at one electrode, often at L2 (birds 1, 2, 5, and 6; Figure 4A). The dominance of information flow into L2 is opposite to the hypothesis (confirmed by onset timing) that neural signals in the auditory pathway have a net flow away from L2 into higher-order areas and is inconsistent with the knowledge that L2 is the main auditory input into the cerebral part of the pathway [22,25,29]. Although this tendency to find interactions to L2 indicates that PDC can be consistent across birds, many of the features recovered do not represent known anatomy.

### Properties of Songbird Auditory Information Flow Networks

**Figure 5:**
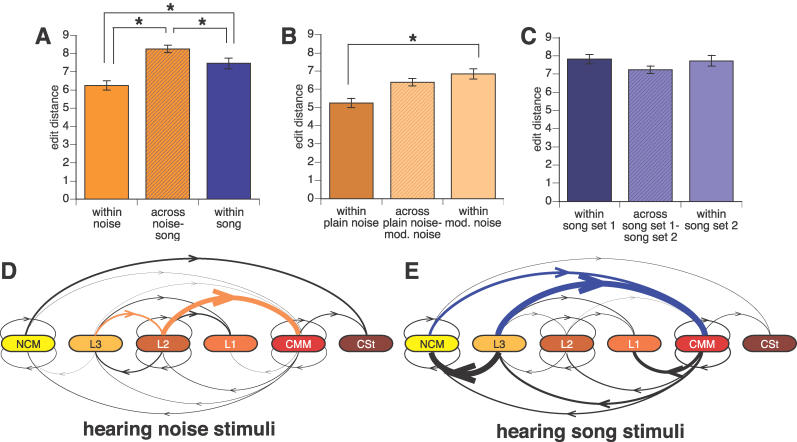
Neural Information Flow Differences due to Hearing Different Kinds of Stimuli (A) Edit distances of networks generated from noise and song stimuli, (B) from plain noise and amplitude-modulated noise, and (C) from two different sets of songs. Error bars represent standard errors of the mean. Asterisks (*) indicate Bonferroni-corrected significance at α = 0.05 (Table S3). (D,E) Differences in information flow between hearing noise and song stimuli, mapped onto the consensus neural flow network of Figure 2B. Colored lines, present more often for the indicated stimuli in at least *n* − 1 of *n* ≥ 4 birds, with no birds showing opposite preference. Line thickness is proportional to the square of the ratio of presence in noise over song for (D) and in song over noise for (E), averaged across all six birds. The line from L3 to NCM in (E) is set to a maximum thickness, as it had an extreme ratio in one bird for song stimuli.

Given that our DBN algorithm is successful at recovering networks that appear to represent neural flow over anatomical paths, we tested whether we could use it to reveal new insights into how the songbird brain processes auditory stimuli. We asked if information flow networks differ when birds process different groups of auditory stimuli. We did this by comparing edit distances (see Methods) *across* two groups to edit distances *within* each group. Edit-distance analysis revealed significant differences between inferred information flow networks produced from data collected while birds were listening to noise stimuli versus song stimuli (Figure 5A; statistics in Table S3). Breaking apart the noise group, we found no significant difference in networks produced across hearing plain white noise versus amplitude-modulated white noise (Figure 5B; Table S3). Breaking apart the song group into two groups of networks produced by two arbitrarily chosen groups of songs, as a control analysis, we would not expect to find significant differences across the groups, and we did not (Figure 5C; Table S3). Thus, the inferred information flow networks had significantly different topologies across noise and song stimuli, but not across different types of noise. However, for both these comparisons there were differences in the variability *within* groups. The song stimuli generated more variability (higher edit-distance values) within networks than the noise stimuli (Figure 5A; Table S3). Similarly, the amplitude-modulated noise generated more variability within networks than the plain white noise (Figure 5B; Table S3). These differences could be due to the fact that the networks in the more variable groups were produced by a greater number of distinct stimuli as well as greater relative differences among those distinct stimuli: one plain white noise stimulus versus four modulated noise stimuli with variation in amplitude (Figure 5B); these five noise stimuli versus eight songs with variation in both amplitude and frequency (Figure 5A). Variation was found (edit distance = 5.2 ± 0.6; mean ± SE) among networks produced from the same plain noise stimulus over four days (Figure 5B). This variation is not due to variability in the heuristic search component of the DBN inference algorithm, as the same dataset run ten times gives the same result (unpublished data). The variation could thus represent any combination of small differences over days, such as neural habituation to familiar sound stimuli [34], variability in the exact location of the electrodes due to slight movement across days, or inherent variability in neural flow networks in response to the same sound stimulus at different times.

Edit distance only measures aggregate differences between information flow networks, so we need other analyses to isolate the specific differences themselves. Examining overall network topology, there was no individual interaction that was differentially present or absent in networks recovered from noise versus song stimuli. However, when calculating the proportion of links which occurred in response to different stimulus presentations, we found that noise stimuli were accompanied by higher probability of inferred information flow among L3, L2, and CMM (Figure 5D), whereas song stimuli were accompanied by higher probability of inferred information flow among NCM, L3, and CMM (Figure 5E). These differences were in the posterior to anterior direction, where inferred flow from L3 to L2 and from L2 to CMM was prominent when hearing noise (Figure 5D, orange lines), and flow from NCM and L3 to CMM was prominent when hearing song (Figure 5E, blue lines). These results suggest that after being processed in L2, noise stimulus signals are preferentially passed directly to CMM, whereas song stimulus signals are preferentially processed in L3 and NCM before being passed to CMM (Video S1).

## Discussion

To our knowledge, our results represent the first demonstration of an algorithm for inferring neural information flow networks that can handle the nonlinearity of real brain electrophysiology data and, in particular, the first use of a Bayesian network inference algorithm for inferring neural information flow networks. Our results also represent the first systematic validation of an inference algorithm's performance against known anatomy. We find that our DBN algorithm infers networks that represent plausible information flow networks: the inferred neural flow is appropriately constrained to the anatomical connectivity network, it matches physiological features of the expected flow network, and it is consistent with the measured temporal dynamics of the system. Since the auditory system of songbirds in which we have performed this validation follows a basic design of avian sensory systems and has homologous/analogous properties with mammalian sensory systems [35,36] we believe our DBN algorithm can be expected to work similarly in other systems and other species.

In contrast, the results of the PDC algorithm are strikingly different. It produces networks with multiple links that are in conflict with known anatomical connectivity. We believe this large difference in performance is due to the fact that a discrete DBN can represent linear or nonlinear relationships between neural activities, whereas PDC assumes that these are linear, in contrast to the fact that these relationships are known to be nonlinear [8,9] (the nonlinearity of our data is shown in Figure S6). This belief is supported by previous results from a simulation study of a different linear method which suggested that applying linear methods to nonlinear interactions results in poor recovery of causal relationships [5]. Other existing linear methods are likely to perform similarly since they are based upon the same foundations as PDC [8–13]. The multiple false positives found with PDC, particularly in the bird with recordings from the meninges, could be due to PDC's tendency to produce false positives from unconnected variables [33]. It is possible that improvements to the linear methods, as well as alterations in the processing of the electrophysiology data, may result in recovery of more accurate neural flow networks. However, linear methods will still be limited by the assumption of linearity.

Recently, within the physical science community as well as in neuroscience, attention has turned to a number of additional frameworks for modeling nonlinear interactions such as dynamic causal modeling, nonlinear extensions of both multivariate (vector) autoregressive models and Granger causality, and transfer entropy [37–43]. However, at this time many of these are restricted to pair-wise interactions, a particular form of nonlinearity, and/or require predefinition of a structure or set of structures to be evaluated, which are not limitations for our DBN approach. Nevertheless, these methods may prove fruitful for use in conjunction with DBNs or as bases for extension to nonlinear inference of information flow networks. For example, one might be able to use a DBN to find the network structure and a dynamic causal model to reveal changes in connection strengths. However, these methods remain to be tested against known anatomy or on neural systems in some cases.

Our DBN inference algorithm is not likely to be recovering perfect networks; as with all statistical inference approaches, the networks we generated are statistical approximations of the true neural flow networks, and as approximations they are not likely to be perfect. However, because they are significantly more biologically plausible than the linear and pair-wise methods that have been utilized to date, the algorithm seems to be the most useful method to date for generating testable hypotheses about how information flows among populations of neurons during various information processing tasks. 

Nevertheless, challenges remain. The microelectrode array we developed had only eight electrodes, but in animals with larger brains like monkeys arrays of hundreds of electrodes have been used [44], and it seems inevitable that larger arrays will be available in the future for songbirds as well. Inference of DBNs over large numbers of variables poses two challenges, one statistical and the other computational. Statistically, the number of variables is not *per se* problematic. The Bayesian scoring metric used is influenced not by the total number of variables in the network, but locally by the number of parents of a variable. Increasing the number of variables increases the search space for the algorithm, but does not influence the statistical power of the score. In this regard, we know from simulation studies that our DBN algorithm scales well with an increasing number of variables and explores the search space sufficiently to accurately learn networks with hundreds of variables [14,17], surpassing the largest electrode arrays that have been used in the brain to date [44]. The ability to statistically infer accurate networks depends primarily on the interaction density of the network (i.e., the number of parents of each variable), which is likely to be high in neural systems but depends somewhat on the level of abstraction one adopts (e.g., multi-unit versus single-unit). When the interaction density increases, more data will be required for accurate inference, as is the case with all statistical inference algorithms. Fortunately, immense amounts of data are available in this setting. Computationally, both the handling of large data amounts necessary for more accurate inference of dense networks and the exploration of large search spaces with thousands of variables can lead to challenges, perhaps necessitating large storage devices and cluster computing. 

Another challenge may be finding the appropriate sampling interval and number of discretization states for different systems being studied. In particular, while a sampling interval of 5 ms may be a critical choice for most cortical networks, if physical distances become larger as in cortical–spinal networks, sampling intervals may need to be increased or higher-order Markov DBNs considering influence from measurements spanning more than one sampling interval into the past may be needed. 

Finally, an open question is whether the recovered networks can be said to be causal, in the sense that one variable interacts with and directly affects the state of the other. Some of the predictions of inference algorithms have been experimentally verified [19,21], and the networks recovered by inference algorithms are sometimes called causal [16,45,46]; a large body of literature exists on the subject of causal inference. However, direct causality is hard to guarantee in situations where not all relevant variables are measured, as is true in our setting; when intervening variables are unmeasured, indirect connections can be recovered [23]. Consequently, we believe that some but not all of the interactions in the recovered neural flow networks are direct causal interactions. Since we did not find interactions over the four paths among auditory regions that do not exist in the anatomy and since the absence of invalid links occurred highly significantly above chance, we can reasonably suppose that our DBN algorithm avoids many, if not all, indirect interactions between brain regions.

Despite the challenges, the inferred neural information flow networks we recover make useful predictions about the biological properties of the auditory pathway. They suggest the dominant direction of putative flow is centrally away from field L sites onto higher auditory areas farther removed synaptically, consistent with findings of hierarchical processing in the auditory system [22,25,29,47]. In hierarchical processing, sounds are sequentially processed from the most simple to the most complex: L2 acts as a processing unit for general sound information and as a filter before sending sound information on to appropriate higher areas that further process ethologically relevant information, resulting in flow differences of the kind we found in the processing of songs versus noise. The differences we observed suggest preferential processing from NCM and L3 into CMM when perceptually discriminating species–specific songs versus other stimuli. These results complement findings that show that NCM and CMM have more complex auditory firing–response properties, selective long-term neural physiological memories, and selective gene regulation, when songbirds hear songs as opposed to synthetic stimuli [24,48–50]. Our results further suggest that CMM may be the highest processing area in the hierarchy.

It is also possible that the algorithm may infer different scales of neural flow networks when applied to different types of neural data. Although we applied our DBN inference algorithm to multi-unit instead of single-unit data due the technical difficulties of obtaining the latter from awake and moving animals, we expect our algorithms to be applicable to single-unit data. We also expect the algorithms to be applicable to diverse human neural data: from microelectrode arrays [51], surface EEG recordings, or fMRI. This is supported by a previous workshop study [46] that has recently come to our attention, where the authors used an approach with similar theoretical underpinnings on human fMRI data to predict cortical networks involved in processing optic flow patterns (without validation of the networks against known anatomy).

Our findings have implications for understanding interactions at multiple levels of biological organization. Just as microelectrode arrays have stimulated the development of computational inference algorithms for understanding brain electrophysiology, the advent of gene expression arrays has led to computational algorithms for elucidating transcriptional regulatory networks [52,53]. The tasks of recovering networks of transcriptional regulation and neural information flow share a similar goal: to infer possible causal relationships between variables on the basis of statistical structure in the observed values of those variables. Indeed, our DBN inference algorithm was first developed for use with time-series gene expression data to infer transcriptional regulatory networks [16,53]; and without further modification, the same algorithm was also effective at inferring neural information flow networks. Provided that sufficient data are available, DBN algorithms can be applied to a wide range of systems at multiple spatial and temporal scales, and may also be effective at recovering networks linking different levels of biological organization [14,54], such as our test with sound and electrophysiology data. In summary, we believe that DBN inference algorithms will prove to be a powerful tool for understanding how the brain perceives the environment and produces behavior, for systems biology more generally, and for any discipline in which the causal interaction structure among large sets of observable variables needs to be inferred.

## Materials and Methods

### Electrophysiology and auditory stimuli.

Detailed electrophysiology methods are described in Protocol S1. In brief, a linear array of eight fluorescently labeled microelectrodes was chronically implanted into cerebral auditory regions of each of six female zebra finches *(Taeniopygia guttata)* (Figure 1A). A set of four stimuli was played to the birds as described in the results. Each stimulus consisted of 1.67–2.17 s of sound, preceded and followed by the same duration of silence (5–6.5 s total). The order of the four stimuli in each repetition of the set was randomized. The plain white noise stimulus was the same across days; the songs and amplitude-modulated noise were different, to prevent measuring long-term electrophysiological habituation changes to familiar natural sounds [24]. During stimulus presentation, multi-unit neuronal voltages were recorded from the eight electrodes (Figure 1B). The data were captured and stored using custom-written virtual instruments in LabView 6.0i (National Instruments, http://www.ni.com), digitized at 20 kHz, band-pass filtered between 0.22 and 5.9 kHz to capture spike activity, and divided into 5-ms time bins. The voltage levels within each 5-ms bin were converted to a single RMS voltage over the whole interval (range 0–0.43 mV); RMS was used to estimate the average magnitude of a voltage signal that fluctuated both above and below zero. This produced 995 to 1,296 data points per stimulus presentation (stimuli ranging from 5–6.5 s due to variations in song length) for the entire silence–sound–silence stimulus. At the end of the playback experiments, the birds were sacrificed, the microelectrode arrays removed, the brains frozen, and 10-μm tissue sections examined under a fluorescence microscope to determine the anatomical location of fluorescently labeled electrode sites. All animal procedures were approved by the Institutional Animal Care and Use Committee of Duke University.

### Data processing.

Before being passed to our DBN inference algorithm, the continuous RMS voltage data were discretized into three levels using a quantile-based method: the lowest 1/3 of values were labeled state 0, the middle 1/3 were labeled state 1, and the highest 1/3 were labeled state 2, i.e., these categories correspond to low, medium, and high RMS values (Figure 1B). Our DBN algorithm requires discretized values, because it uses a discrete BN approach. We chose three-state discretization, as this was typically optimal with our algorithm [16].

### Dynamic Bayesian Network inference algorithm.

The DBN inference algorithm used in this paper is written in C++ [16], is modeled on an algorithm called NetworkInference [14,53], and consists of four elements: i) a first-order Markov DBN model of dependence and conditional independence between discrete variables over time, ii) a Bayesian scoring metric to measure how well a particular network represents the observed data, iii) a greedy search procedure with random restarts to identify a network with as high a score as possible (the inferred network), and iv) an influence score which assigns a putative strength and sign (+ / −) to each of the dependencies in the inferred network. The inferred network, thus, represents dependence relationships, along with their sign and magnitude, between activity levels in the measured brain regions. For a particular link, this means that the activity level in one region is useful for predicting the activity level (at a later time) in another region; we interpret this biologically to mean neural information flow from the first to second region is responsible for this statistical relationship. Because we use a first-order Markov DBN, variables can only be directly affected by variables at the previous time step. The captured relationships may be of any aribitrary combinatoric shape and thus are not restricted to linear nor additive forms. The direction of the relationships is determined using the time-dependent feature of DBNs, such that relationships flow only forward in time. More details on the algorithm are available in Protocol S1, as well as in Figures S7 and S8. We later developed a more flexible, efficient, and user-friendly program in Java called Banjo (Bayesian network inference with Java objects). It can be licensed free for noncommercial use and is available along with complete source code from http://www.cs.duke.edu/~amink/software/banjo/. The C++ version is available upon request.

To confirm that it is reasonable to use a single top network from the search to estimate the statistical dependencies present in the data, we examined the top 10 networks found for four example datasets. We found that the links present in the single top network were present in 9.5 ± 0.2 (mean ± SE) of the top 10; whereas links found in the top 10 but not present in the single highest-scoring network were found in only 2.1 ± 0.3 of the networks. This indicates that (1) there is no evidence of a highly different structure that scored similarly, and (2) the top network represents a consistent summary of the dependencies, where the other high, but lower-scoring networks have small and different variations on the same structure.

### Neural information flow network inference.

We passed the discretized RMS values from the 20 repetitions of one stimulus on one day to our DBN inference algorithm. This provided 19,000–25,920 data points for each network inference task, well above the 2,000 data points we determined through simulation studies to be sufficient for highly accurate inference [16]. For each dataset, the algorithm took ∼5–10 min using a single Dell PC with a 2.26 GHz CPU and 1 GB RAM to find a high-scoring network. Because not all networks generated for each bird were identical, not even those from the same stimulus, to determine the most robust features over different datasets, we generated summary networks for each bird (Figure 2A). These contained interactions that occurred with highly significant consistency, having repetition across the individual networks in greater than the 99th percentile of a Monte Carlo analysis (see the section Statistical analyses). These interactions were used to generate a consensus network across birds (Figure 2B).

### Data randomization.

For testing the DBN algorithm with random data, three different types of random data were produced from two datasets of each bird: 1) randomly shuffled RMS values within each electrode's data across all 20 repetitions of a stimulus, destroying the temporal relationships among electrodes but maintaining the descriptive statistical properties of each electrode, 2) uniformly distributed random values within the range of the real RMS data for each electrode, and 3) random values within the range of and having the same first-order Markov properties as the discretized RMS data for each electrode, but no correlation across electrodes. For the third, we produced simulated random data using a Bayesian network with the data from each variable *X_i_* dependent on the immediately previous time point. We used the conditional probability P(*X_i_*(*t*)*|X_i_*(*t* − 1)) estimated from the real data, and had no interaction between the variables. For testing PDC with random data, we used the first two types of random data, using the same random data we produced for testing the DBN. The third type of random data is unique to discrete data and so was not used in evaluating PDC. We additionally made further random data of the second type for PDC, using all the datasets from two birds to further investigate possible false positives.

### Link length.

We determined the number of links of each length (1–7 electrodes distant) in the 16 (or 12) networks for each bird, with lengths based on the electrodes' linear anatomical order. We divided link lengths by the maximum number of links of that length possible, to normalize these values (for example, with eight linearly arranged electrodes, a maximum of 14 directed links of length 1 can be recovered, but only two of length 7). Only lengths 1–6 were included in the statistical analysis because the most distant electrodes for bird 3 were only of length 6. Due to the missing electrodes, this bird had a different distribution of possible link lengths, which was taken into account.

### Edit distance.

The edit distance between two networks is defined as the minimum number of edit operations (insertions and deletions of links) needed to transform one network into the other. The resulting number is a measure of how well two networks agree, both on the links that exist and the links that do not exist; it is equivalent to the number of links that differ between the two networks. Because these are dynamic networks, links differing in orientation are unambiguously distinct, and two edits are required to turn one into the other. We calculated three sets of edit distances among the networks for each bird: 1) the distances between all pairs of networks within one group, 2) the distances between all pairs of networks within the other group, and 3) the distances between all pairs across groups, consisting of one network from one group and one from the other group. The average of these edit distances is a measure of the amount of variation found either within networks of a group (1 and 2) or across the networks of two groups (3). Because each of the six birds had different electrode locations, edit distances could not be calculated between networks from different birds. Thus networks were only paired within single birds.

### Statistical analyses.

To determine which links were recovered significantly across networks for each bird, we performed a Monte Carlo analysis. The 16 (or 12 for bird 1) recovered networks per bird were used to generate 1,000 sets of 16 (or 12) random networks per bird with the same variables and number of links as the recovered networks. For each of the 1,000 sets of 16 (or 12) random networks per bird, we counted the number of times each of the possible 56 links among eight variables (30 links among six variables for bird 3 missing two electrodes) occurred within the set. Because in random networks each of the 56 (or 30) links is equivalent, we combined the occurrence counts of all links to produce a distribution of occurrence counts within a set by chance. From this distribution for each bird, we calculated the minimum number of times a link would have to occur within the original 16 (or 12) recovered networks to be above the 99th percentile of the distribution of chance occurrences (*p* < 0.02 for a two-tailed test). Only those interactions meeting this 99th percentile criterion (occurrence in at least 44%–63% of the networks, depending on the bird) were considered significantly consistent. This same Monte Carlo analysis was used to calculate significance values for avoidance of links representing connections known not to exist in the anatomy. We produced distributions of the occurrence counts of links for such nonexisting connections expected randomly across all six birds, by summing the number of links found between all electrodes of each nonexisting connection for the 16 (or 12) random networks across all six birds, for each of the 1,000 random generations. This distribution was used to calculate a *p*-value for finding links in as few or fewer networks than the actual number of times each link occurred (one-tailed for the directional hypothesis). A hypergeometric test was used to calculate the probability of finding all inferred interactions matching known anatomical paths. A hypergeometric test provides the probability of drawing a particular number of successes and failures given the size of a sample drawn at random (without replacement) and the total number of possible successes and failures in the population. In our case, we looked at the probability of getting the number of interactions we found matching known anatomy (successes) and no interactions violating known anatomy (failures), based on the total number of anatomically possible paths and paths known not to exist. We looked only at those interactions and possible paths that could be categorized as known connections or known nonconnections, i.e., excluding those of auditory regions with CSt and those between electrodes in the same brain regions.

When comparing the values of a given variable (e.g., proportion of links found) across different conditions (e.g., links of different lengths), we used multiple-way analyses of variance (ANOVA). When the data lent themselves to it, we used repeated-measures ANOVA, which compares variables measured from the same subject multiple times (e.g., one bird listening to several stimuli). We used bird identity as a factor to statistically control for possible differences among birds. Pair-wise post-hoc multiple comparisons were performed for significant ANOVAs using Bonferroni-corrected significance values. Edit-distance statistics were performed using an ANOVA and pair-wise multiple post-hoc tests. Thus, two groups of networks would be considered significantly different only if both an ANOVA shows a significant difference between the three sets of distances and the mean distance between groups is significantly greater in pair-wise tests than both mean distances within either group.

### Partial directed coherence.

We obtained the Matlab code for the PDC algorithm [11] from its creators, Drs. Baccalá and Sameshima at the University of São Paulo, Brazil. We applied PDC to our undiscretized RMS voltage values using options corresponding to the normalized PDC of Equation 18 in [11]. Our datasets had features, such as noncontiguous repetitions and large changes in firing rate, with which PDC has difficulty dealing. We corresponded with Drs. Baccalá and Sameshima to determine the optimal method to apply PDC to our datasets and learned that 1) because PDC cannot handle sections of noncontiguous data in a single analysis, we needed to perform PDC analyses on each of the 20 repetitions of a stimulus during one day separately, and 2) because PDC requires stationary data recorded from a single steady state, we needed to perform PDC analyses on each 1/3 subsection of the silence–sound–silence stimuli separately. This led to a total of 60 analyses per dataset (20 repetitions of the three subsections).

PDC does not provide a method of combining multiple analyses to produce a single network representing the neural information flow inferred from the dataset. We needed a single network per dataset to compare the PDC with the DBN analysis. To generate one, we expanded upon the heuristic threshold PDC uses to determine links between variables, namely, a link is considered to exist between two variables if the maximum PDC across all frequencies is greater than 0.1 [33,55]. We extended this reasoning as follows: if the average of the maximum PDC between two electrodes of the 20 repetitions was greater than 0.1 for any of the 1/3 subsections, this is considered a link for that stimulus presentation. This produced one network for each stimulus presentation, equivalent to the output of our DBN. From this point forward, we used the same Monte Carlo method as before to determine interactions recovered significantly across the 16 (or 12) stimulus presentations. We also examined the effect of removing analyses where either of the two diagnostics provided by PDC indicated a poor fit of the model (*Passk* ≥ 0.1 or *Portk* = 0). This was performed before determining the average of the maximum PDC over 20 repetitions. Subsequent steps were unchanged.

## Supporting Information

Figure S1 Electrophysiological Recordings (A) Female zebra finch with implanted electrodes.
(B) Removed skull of one bird showing below it the eight implanted electrodes and ground wire.
(C–E) Approximate locations of all electrodes in all birds. Drawings represent sagittal sections at (C) ∼0.4 mm, (D) ∼0.6 mm, and (E) ∼0.8 mm from the midline. Electrode locations from different birds are indicated with different symbols. The lateral striatum (LSt) electrode of bird 5 was in a plane further lateral than our drawings. The CSt electrode of bird 2 is in a plane between (D) and (E). The front of the brain is to the right and the dorsal part is to the top. Abbreviations are as in Figure 1, with additional terms: S, septum; HA, hyperpallium apicale; HF, hippocampal formation; M, mesopallium.
(893 KB PDF)

Figure S2 Significant Interactions When Measurements from All Six Birds (Each Colored with a Different Color) Were Pooled Together (46 Electrodes) along with One Node Representing the Sound Stimulus: A Binary Variable Indicating the Silent versus Sound Portion of the Stimulus Bird 1 is red, 2 purple, 3 grey, 4 yellow, 5 pink, and 6 green. Numbers next to each link represent the number of times it repeated across the 12 networks (as one bird had data from only three days, this analysis was done using only these three days for all birds), and link thickness is scaled to the square of this value. As can be seen from the figure, no links were found between electrodes in different birds, and no links were found to the sound stimulus variable.
(380 KB PDF)

Figure S3 Networks Inferred from Subsections of Data across All Stimuli, in Comparison with Entire Stimuli, for Two Example Birds Consistent interactions are shown as described in the legend of Figure 2A.
(123 KB EPS)

Figure S4 Proportion of Links of Each Length Found Relative to the Maximum Number of Links of That Length Possible There was a significant difference in the proportion of links of each possible length, with shorter links predominating (statistics in Table S2). The length of a link is defined as the electrode number difference between two pairs of electrodes. Error bars represent standard errors of the mean.
(16 KB EPS)

Figure S5 Response Latencies to Hearing Sound across Different Brain Regions Simultaneously Recorded Plotted are mean latencies to response onset for each brain region. Response onset was defined as the time of the first of five consecutive 1-ms bins in which the RMS response was three standard deviations above baseline activity (using the responses from four different sessions in which white noise was used as an auditory stimulus). Error bars represent standard errors of the mean. Additional details will be published separately (TVS and EDJ).
(79 KB PDF)

Figure S6 Nonlinear Relationships between Electrodes Shown are the RMS values from all eight electrodes of bird 4, plotted against each other, using data from the 20 repetitions of modulated white noise. Note that all the scatter plots have a wide range of relationships, not following any particular line (i.e., they are nonlinear). The two details show electrodes with either two distinct relations (blue) or a broad range of relations (red).
(147 KB PNG)

Figure S7 Dynamic Bayesian Network in the Auditory Pathway A putative causal network of neural information flow (top) can be represented as a DBN at two time slices 5 ms apart (bottom). Discretized RMS values at all electrode locations at time t + 5 ms in the DBN are predicted by both themselves and their putative inputs at time t. Note a cyclic interaction between L3 and L2 is represented by acyclic interactions across time in the DBN.
(52 KB EPS)

Figure S8 DBN Inference Algorithm The algorithm uses a greedy search with random restarts to find an inferred network with a high score. Then, influence scores are calculated for all links in the inferred network. Numbers denote order of steps.
(118 KB EPS)

Protocol S1 Supporting Methods (89 KB DOC)

Table S1 Statistics for Analysis of Subsections of Stimulus from Main Text and in Figure 3B and 3C (62 KB DOC)

Table S2 Statistics for Comparison of Proportion of Links of Each Length in Figure S4
(51 KB DOC)

Table S3 Statistics for Edit-Distance Comparisons in Figure 5A–5C (57 KB DOC)

Video S1 Interpretation of Dynamic Neural Flow Based upon the DBN Recovered Networks and Known Biology (47 KB PPT)

## Abbreviations

ANOVA: multiple-way analyses of variance
DBN: dynamic Bayesian network
PDC: partial directed coherence
