# Supplementary figures and images for: Computational Inference of Neural Information Flow Networks
Source: PLoS Comput Biol. 2006 Nov 24;2(11):e161. doi: 10.1371/journal.pcbi.0020161 (PMC1664702; doi:10.1371/journal.pcbi.0020161)

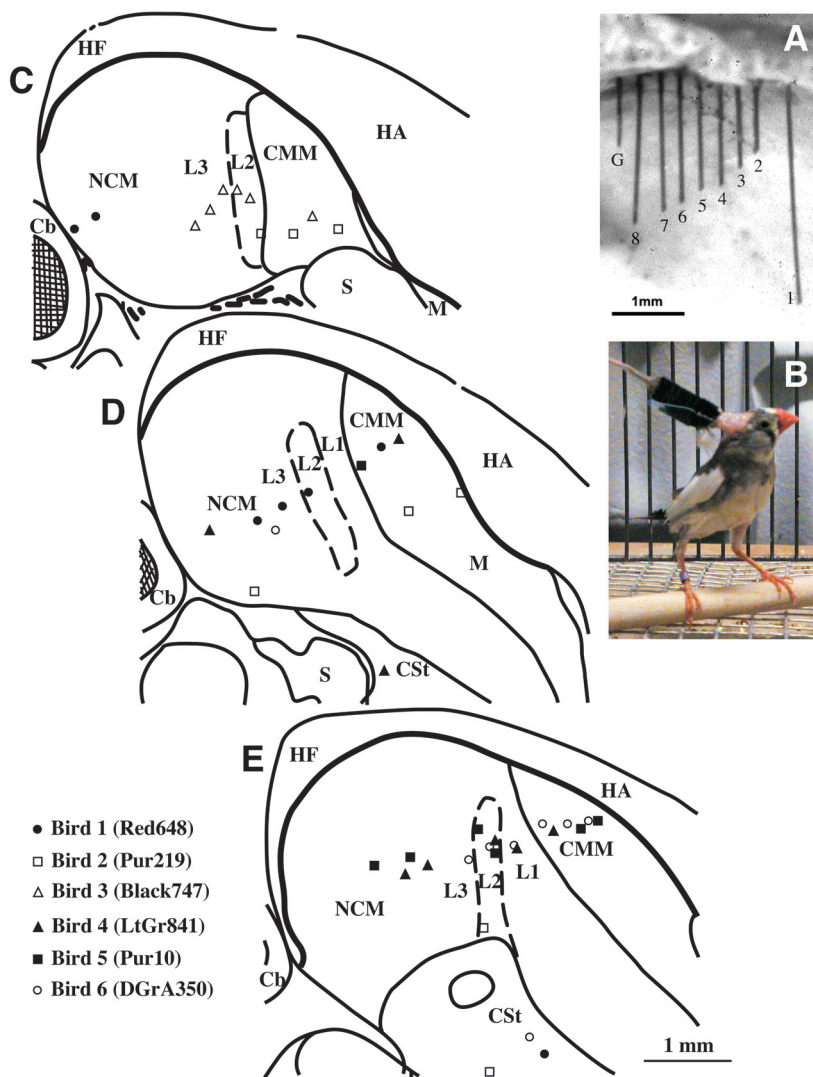

Supplement: Figure S1 — (A) Female zebra finch with implanted electrodes. (B) Removed skull of one bird showing below it the eight implanted electrodes and ground wire. (C–E) Approximate locations of all electrodes in all birds. Drawings represent sagittal sections at (C) ∼0.4 mm, (D) ∼0.6 mm, and (E) ∼0.8 mm from the midline. Electrode locations from different birds are indicated with different symbols. The lateral striatum (LSt) electrode of bird 5 was in a plane further lateral than our drawings. The CSt electrode of bird 2 is in a plane between (D) and (E). The front of the brain is to the right and the dorsal part is to the top. Abbreviations are as in Figure 1, with additional terms: S, septum; HA, hyperpallium apicale; HF, hippocampal formation; M, mesopallium. (893 KB PDF) [file pcbi.0020161.sg001.pdf]

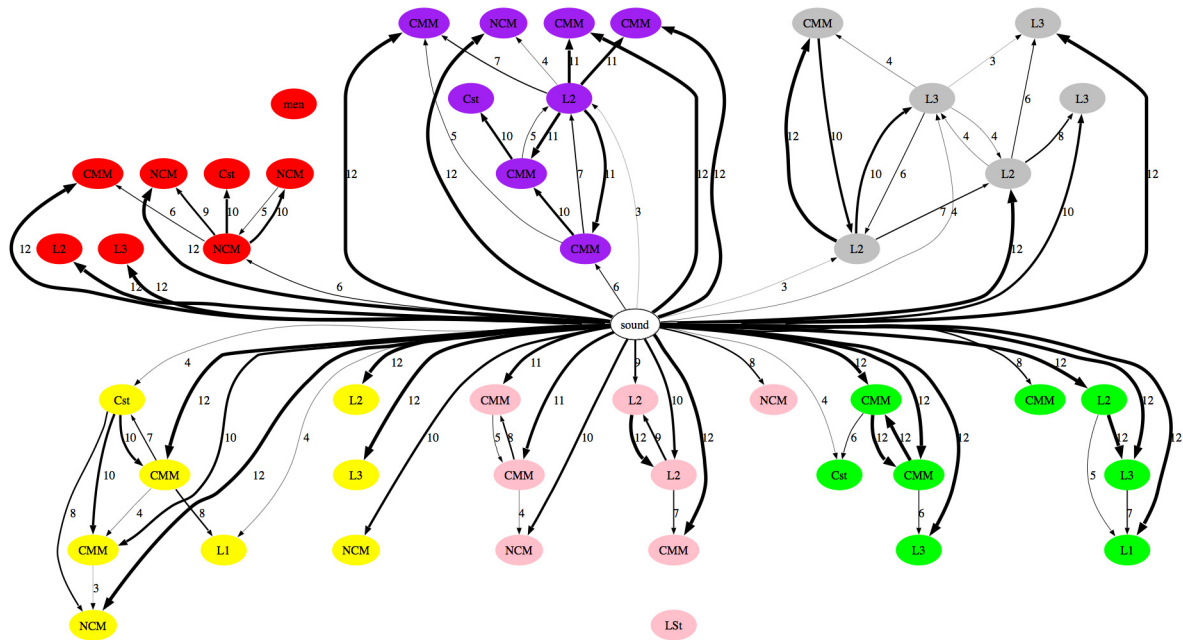

Supplement: Figure S2 — Bird 1 is red, 2 purple, 3 grey, 4 yellow, 5 pink, and 6 green. Numbers next to each link represent the number of times it repeated across the 12 networks (as one bird had data from only three days, this analysis was done using only these three days for all birds), and link thickness is scaled to the square of this value. As can be seen from the figure, no links were found between electrodes in different birds, and no links were found to the sound stimulus variable. (380 KB PDF) [file pcbi.0020161.sg002.pdf]

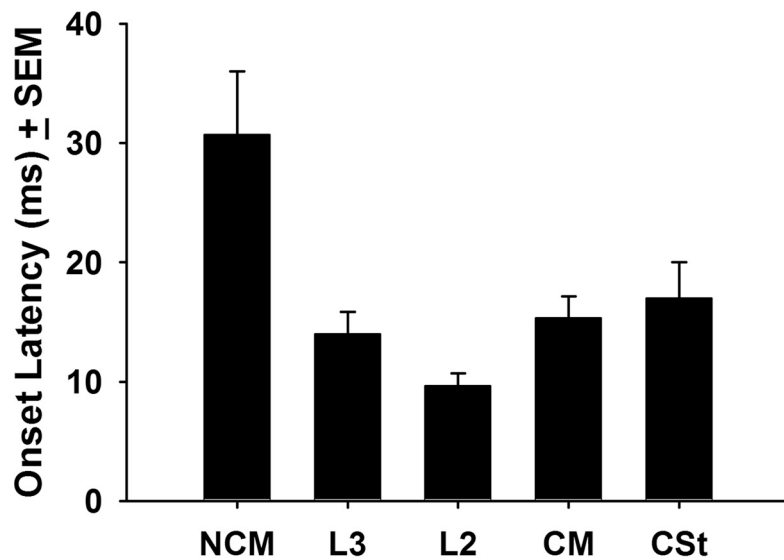

Supplement: Figure S5 — Plotted are mean latencies to response onset for each brain region. Response onset was defined as the time of the first of five consecutive 1-ms bins in which the RMS response was three standard deviations above baseline activity (using the responses from four different sessions in which white noise was used as an auditory stimulus). Error bars represent standard errors of the mean. Additional details will be published separately (TVS and EDJ). (79 KB PDF) [file pcbi.0020161.sg005.pdf]

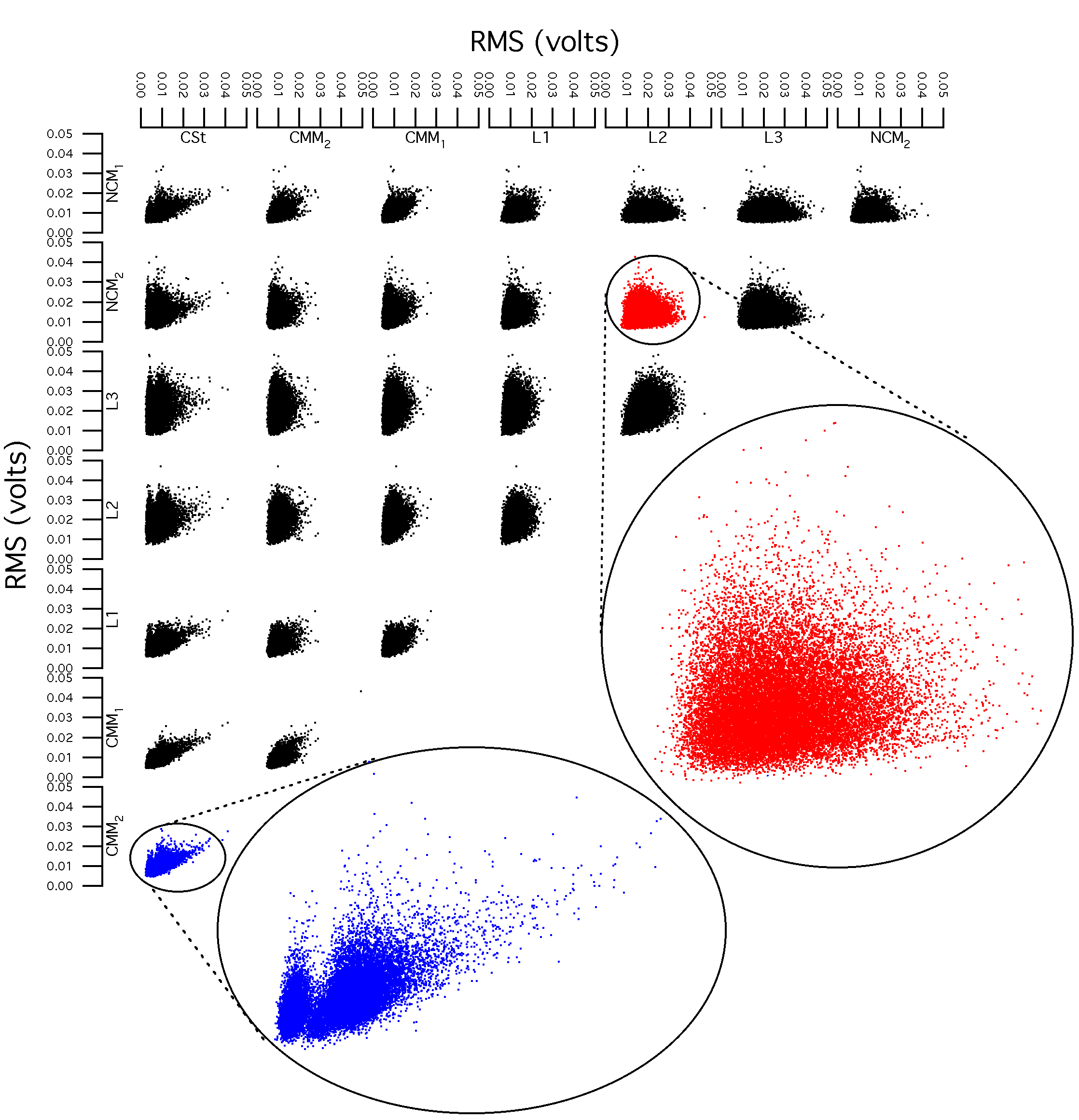

Supplement: Figure S6 — Shown are the RMS values from all eight electrodes of bird 4, plotted against each other, using data from the 20 repetitions of modulated white noise. Note that all the scatter plots have a wide range of relationships, not following any particular line (i.e., they are nonlinear). The two details show electrodes with either two distinct relations (blue) or a broad range of relations (red). (147 KB PNG) [file pcbi.0020161.sg006.png]
