# Supplementary material for: Computational Inference of Neural Information Flow Networks
Source: PLoS Comput Biol. 2006 Nov 24;2(11):e161. doi: 10.1371/journal.pcbi.0020161 (PMC1664702; doi:10.1371/journal.pcbi.0020161)
Supplement: Protocol S1 — (89 KB DOC) [file pcbi.0020161.sd001.doc]

PROTOCOL S1

SUPPORTING METHODS

## **Electrophysiology Experiments**

## i) Electrode arrays and surgery

To a nine-channel nano-connector (Omnetics Corp., Minneapolis, MN), we attached 8 blunt-cut isonel-insulated tungsten microwire electrodes (50 μm diameter; impedance approximately 0.8 Mohms, range: 0.6-1.2). The electrodes were positioned in a straight line. The rostral-most electrode (electrode 1) was situated ~375 μm in front of the next electrode (electrode 2), followed by others each spaced ~250 μm apart. The electrodes were cut in such a way that electrode 1 was the longest and electrodes 2-8 were the shortest to the next longest, at an angle of 30º from the horizontal (Fig. S1A). This configuration ensured that during surgery electrode 1 was aimed at CSt, while the other seven covered the stretch from CMM through L1, L2, L3, to NCM. A ninth electrode was added either behind electrode 8 or in front of electrode 1 as a ground (with a large section of insulation removed; in birds 1-4) or as a reference electrode (similar to the other electrodes, but implanted in a non-auditory brain region anterior to CMM; in birds 5 and 6). In the latter case, a separate silver wire was used as the ground electrode. This set-up (with separate reference and ground electrodes) significantly reduced the amount of movement artifact.

Before implant, fluorescent DiI (red) and DiO (yellow) dyes (Molecular Probes, Eugene, OR) were applied and dried to alternating electrode tips in the array, such that their locations in the brain could later be identified. The dyes did not interfere with the electrode recordings nor with the types of responses the neurons gave when compared to a control implant that had electrodes without dyes (see also [1,2]). The arrays were implanted under isoflurane anesthesia. Birds were fixed in a custom-made stereotaxic frame (H. Adams, CalTech) with the head held at a 45^o^ angle. On one side of the brain, a rectangular opening was made in the skull above the auditory areas and the array was lined up parallel to the midline of the brain. Electrode 1 was zeroed to the split in the mid-sagittal sinus (visible through the bottom layer of the skull), maneuvered 2.75mm rostral, 0.45-0.7 mm lateral (slightly different coordinates in different birds), and then lowered to ~2.7mm below the surface, with the other electrodes following in their pre-configured positions. Every time an electrode almost touched the *dura mater*, a small slit was cut into the *dura* to allow the electrode to slip in. As the electrode array was advanced, electrophysiological activity on each electrode was recorded using an extracellular amplifier (FHC Inc, Bowdoinham, ME) and custom-written software in LabView™ 6.1 (National Instruments, Austin, TX). The LabView software was based upon that developed in the laboratory of R. Mooney (version of F. Livingstone and M. Rosen). To search for auditory responses, the experimenter whistled scales. When at a depth where all or most of the electrodes showed increased multi-unit responses to the whistles, the array was left in place and attached to the skull using dental cement and cyanoacrylate glue. The birds were allowed to wake up and were eating and drinking within 20 minutes. After ~2 days of recovery, they were attached to the recording set-up in a soundproofed anechoic room in a 43 x 60 cm cage.

We chose this stable chronic set-up to obtain multi-unit recordings, instead of a microdrivable one to obtain single units, as we considered anatomical stability across days to be more important. This way, we ascertain the response properties of the same small populations of neurons to a wide range of stimuli, in awake, behaving birds. This would have been impossible with single unit recordings, which would either have required restrained or anaesthetized birds, or would only have allowed recording from the same electrode for short amounts of time (< 1 hour typically). Multi-unit changes are interpreted either as changes in the population response by all individual neurons making up the population responding in the same way or by subsets of neurons changing their response.

All experiments were approved by the Duke University Institutional Animal Care and Use Committee.

## ii) Electrophysiological recordings

The recording room was separated from an anteroom, in which the experimenter was located together with all the recording equipment, by a one-way window. Through this window and a TV monitor in the recording room connected to a SONY DCR-PC100 Digital HandyCam, the bird’s behavior was monitored. The birds had *ad lib* food and water in the cage and the lights were set to a 12/12 L/D schedule. We recorded multi-unit activity through a lightweight recording cable with headstage (Plexon Inc, Dallas, TX), connected to the nano-connector attached to the bird at one end (Fig. S1B), and a motorized commutator (Dragonfly Inc, Ridgeley, WV) attached to the cage at the other end. This cable was connected at all times, and the birds habituated to its presence overnight, after which the experiments were started. Once habituated, the birds moved around freely in their cage, eating, drinking, flying, sleeping and grooming.

The headstage contained one unit-gain op-amp for each channel, including the reference wire, to match the impedance of the wires to the impedance of the amplifier. The reference channel was split into a non-amplified ground channel and an amplified reference channel for birds 1-4, whereas for birds 5 and 6 the connection between ground and reference was cut to reduce movement artifact and a separate groundwire was implanted. The signals from the commutator were sent to a multi-channel extracellular amplifier (500x amplification, band-pass filtered between 220 Hz and 5.9 kHz; Plexon, Inc., Houston, TX) and from there on to a PCI-6071E Analog to Digital conversion card (National Instruments, Austin, TX) in the computer. The data were digitized at 20 kHz using our custom-written LabView software. We also recorded the sound in the recording room using a Sennheiser ME62 microphone, amplified via a Midiman microphone amplifier, fed into the PCI-6071E card, and digitized together with the electrophysiological data, at the same rate.

## iii) Anatomy

In the last recording session, the birds were exposed to 50 repetitions of a novel conspecific song for 15 minutes to induce immediate early gene expression [3]. We then waited another 10 minutes to let the mRNA accumulate before quickly decapitating the birds, dissecting out the brain and fast-freezing it. Brains were stored at -80ºC until processing. Brains were cut on a cryostat into four or six series of alternate 10 μm sagittal sections. One series was stained with DAPI and coverslipped for examination of the alternating fluorescent DiI and DiO electrode tracts. Another series was hybridized with radioactive RNA probe for the immediate early gene egr-1 using a previously established procedure [4] to visualize known gene expression differences in the auditory forebrain areas [5]. We identified L2 by its lower egr-1 levels relative to the surrounding brain areas. Thus, we had four means of anatomically identifying electrode locations: 1) fluorescent dye from the electrode tips; 2) hearing-induced egr-1 gene expression patterns; 3) gliosis along the electrode tracts in some cases, and 4) known cresyl-violet defined boundaries among brain regions. This ensured highly accurate location of recording sites. It also allowed determination of relationships between neural activity and hearing-induced gene expression, which will be reported separately.

Based upon their locations, all electrodes in all birds were found and assigned to one of six auditory forebrain areas (Fig. S1C-E): NCM, Field L3, Field L2, Field L1, CMM, and CSt. To assign the electrodes to these specific brain areas, we looked for the section that contained the electrode tip (deepest point of the tract) and determined its location in the auditory forebrain relative to the different laminae. Since the boundary between L3 and NCM is not easy to determine in Nissl stain or with egr-1, we estimated it based on the distance from L2 (~0.4mm).

## iv) Playbacks

During playbacks, the birds were able to move around freely. This advantage of having more natural behavior came with some loss in control over the animal’s position relative to the sound source. We compensated for this loss in several ways. Firstly, stimuli were played simultaneously from 2 speakers located equidistantly on either side of the cage, placed as far as possible from the cage (approximately 60 cm from the nearest point of the cage), so that any movement of the bird would represent only a fraction of the distance between it and the speaker. Movement away from one speaker would automatically bring the bird closer to the other speaker. Turning the ear contralateral to the recording away from one speaker again would automatically result in turning it towards the other one. Because of this set-up, the movements of the bird do not result in significant changes in the stimulus intensity as they reach the ears of the birds.

Stimuli were encoded as WAV files. Each song stimulus consisted of two motifs, without introductory notes. White noise and modulated white noise were generated using custom-written software in LabView. All stimuli were preceded and followed by a period of silence of exactly the same length as the stimulus itself. We generated similar intensities for all stimuli by playing them through the same speakers as used in the experiment, re-recording them (digitized at 20 kHz) using a microphone with a flat spectral response profile (Radioshack lapel microphone), and then adjusting the volume of the stimulus until all stimuli produced a microphone-recorded signal with the same Root Mean Square (RMS), resulting in a similar average power across stimuli. The speakers were Cambridge Soundworks Creative CSW1500 surround sound gaming speakers connected to a SoundBlaster Live card in the computer and controlled through custom-written software in LabView. The speaker level was set so that a 1 kHz pure tone delivered 80 dB SPL in the center of the cage (Radioshack dB meter).

**Dynamic Bayesian Network (DBN) Inference Algorithm.**

Details on the theory of Bayesian networks and DBNs can be found in Friedman et al. [6] and Heckerman et al. [7]. The interested reader is also referred to Cowell 2001 [8], wherein is described the mathematical equivalence of score-based and conditional independence test-based methods of determining the best Bayesian network to model a data set. The specific software we used was developed by Yu et al. [9] in C++, based upon earlier software developed by Hartemink in C. We later developed a more flexible, efficient, and user-friendly software package in Java called Banjo. Banjo can be licensed free for non-commercial use and is available with complete source code over the web from *http://www.cs.duke.edu/~amink/software/banjo/*. The C++ research-grade version is available upon request. Each of the four elements in our algorithm mentioned in the Methods section of the main text is described below in the context of neural information flow networks.

*i) DBN model*

A DBN is an extension of a static Bayesian network (BN). A BN is a graphical representation of a joint probability distribution over χ, where χ={*X*_1_, ..., *X_n_*} is a set of random variables *X_i_*. A BN is specified as a pair <*G*,Θ>. The variable *G* represents a directed acyclic graph whose vertices correspond to the random variables *X*_1_, ..., *X_n_* (activity levels at one of the individual electrode locations in our case) and whose directed links from *X_i_* to *X_j_* indicate a statistical conditional dependence of *X_j_* on *X_i_*. All variables which have a directed link to *X_i_* are known as its parents [*Pa*(*X_i_*)]; all variables to which *X_i_* has a directed link, and recursively all variables receiving links from such targeted variables and their targets, are known as its descendents. Each variable *X_i_* is independent of its non-descendents in *G* given its parents in *G*. The variable Θ represents the collection of parameters that quantify the probability distributions associated with each variable *X_i_*. Each of these probability distribution parameters is specified as = P(*X_i_=x_i_*|*Pa*(*X_i_*)=*pa*(*X_i_*)): namely, the probability of *X_i_* taking on the value *x_i_* (one of the discretized RMS values in our case) given its parents *Pa*(*X_i_*) having the values in a particular instantiation of the parents, *pa*(*X_i_*), for all *x_i_* and *pa*(*X_i_*). These parameters combine to form the unique joint probability distribution:

P(*X*_1_, ..., *X_n_*) = P(*X_i_* |*Pa*(*X_i_*)).

Note that this probability distribution allows arbitrary combinatoric relationships between parent and child values: each is unique to its parent configuration and child state. This feature allows discrete BNs to model many types of relationships, including nonlinear and nonadditive relationships.

A DBN extends this framework by including the dimension of time. We use a first-order Markov DBN, meaning that we consider variables at one time step to be affected only by those in the immediately previous time step. Such a DBN is a graphical representation of a joint probability distribution over χ', where χ' ={*X*_1(_*_t_*), ..., *X_n_*(*_t_*), *X*_1(_*_t+∆t_*), ..., *X_n_*(*_t+∆t_*)} is a set of discrete random variables *X_i_* measured at both time *t* and time *t+∆t* (*∆t*=5ms in our case). Just like a BN, a DBN is specified as a pair <*G*,Θ>. The graph *G* (an information flow network in our case) is restricted in a DBN so that links are only allowed to go forward in time, i.e., from a variable *X_i_*(*_t_*) to *X_j_*(*_t+∆t_*). Additionally, we require that all variables must have directed links from themselves at time *t* to themselves at time *t+∆t*, i.e., all *X_i_*(*_t_*) link to *X_i_*(*_t+∆t_*). The collection of parameters Θ consist of , as above, for all *X_i_*(*_t+∆t_*) in χ'.

We used a DBN because it has two important advantages over static BNs to model biological systems. First, BNs cannot model feedback loops among variables due to the acyclicity restriction on the graph *G*. However, information flow in the brain can be reciprocal. A DBN allows representation of cyclic interactions between two or more variables over time because it models all variables at more than one point in time: a cycle over time is represented as acyclic interactions between variables across the two times (Fig. S7). Second, the unique joint probability distribution of a BN can sometimes have several different equivalent factorings that differ only in the direction of some links. These graphs form a Markov equivalence class and are indistinguishable probabilistically, leading to uncertainty about the direction of influence between variables. In contrast, in a DBN only one factoring in the Markov equivalence class will match the additional restriction that the direction of links in *G* go forward in time, as is expected with brain function, and thus there is no uncertainty about direction of influence.

*ii) Bayesian scoring metric*

The problem of discovering a DBN (or BN) from a collection of observed data is stated as: Given a data set *D*={*Y*_1_*, ..., Y_m_*} of observed instances of χ, where *Y_k_* represents a vector of values (discretized RMS values in our case) for each *X_i_* (electrode location) in χ at time *k*, find the most probable graph *G* (information flow network) for explaining the data contained in *D*. This is done using a Bayesian score function:

*Bayesian Score*(*G*:*D*) = log P(*G*|*D*) = log P(*D*|*G*) + log P(*G*) – log P(*D*),

which states that the score of the graph *G* given the data in *D* is equivalent to the log posterior probability of *G* given *D*. As we are looking at only one data set *D*, its marginal probability P(*D*) is equal for all *G*, and thus it is not necessary to calculate this singular P(*D*) in order to compare different *G*. This reduces the equation to:

*Bayesian Score*(*G*:*D*)= log P(*D*|*G*) + log P(*G*).

We have no expectation to prefer some information flow networks over others, so the prior over graphs P(*G*) is uniform for all graphs *G* that are valid DBNs and 0 for all that are not; thus, we do not calculate this term as we are only interested in comparing *G* that are valid DBNs. This reduces to solving for only the log of the marginal likelihood P(*D*|*G*). We calculated P(*D*|*G*) using the Bayesian Dirichlet (BDe) score method. This is done by integrating over all possible parameter assignments Θ:

log P(*D*|*G*) = log ∫_Θ_ P(*D*|*G*,Θ) P(Θ|*G*) dΘ.

This integral is solvable, assuming the prior over parameters P(Θ|*G*) is Dirichlet (the conjugate distribution for a multinomial), as:

log P(*D*|*G*)= log (),

where *n* is the number of variables *X_i_* in χ, *q_i_* is the number of parent instantiations *pa*(*X_i_*) of the parents of *X_i_*, *r_i_* is the number of values *x_i_* of *X_i_*, Γ(·) is the gamma function, *α_ij_* and *α_ijk_* are equivalent sample size statistics of the Dirichlet prior distribution, and *N_ij_* and *N_ijk_* are counts in *D* of the number of times the parents of *X_i_* take on instantiation *j*, and the number of times the variable *X_i_* takes on value *k* with parents in instantiation *j*, respectively.

To provide an intuitive understanding how this score relates to the data, we note the following: The rightmost product term, Γ(*α_ijk_*+ *N_ijk_*)/ Γ(*α_ijk_*), is higher when the counts are more concentrated in a particular child value. This indicates that the scores are better when a given parent state is more useful for predicting the child, matching with the definition of statistical dependence. The other product term, Γ(*α_ij_*)/ Γ(*α_ij_* + *N_ij_*), is higher with (a) fewer parent states and (b) equal examples of each state. The feature (a) is the inherent penalty for complexity which prevents over-fitting of the data, and the feature (b) indicates that the score is higher when the data is distributed more evenly across its possible values.

In addition to the actual data, two other factors influence this score: the value used for the Diriichlet prior's equivalent sample size (ess) and the level of discretization of the variables. The effect of the ess is such that a higher value means the prior belief is more strongly that ‘anything can happen’: the score for any interaction is higher and thus it is easier for any link to be found. We used a low ess of 1 to put a large emphasis on the influence of the data for determining which links exist. The effect of discretization is such that more discrete levels can lead to more detailed predictive power from parent state to child state, but it also leads to more parent states and more sensitivity to noise. Because we discretize using a quantile method, it does not lead to more unevenly distributed data across states, although with other discretization methods this could be the case. Since these features affect inference accuracy in opposing ways, we must find a balance between using more discrete levels to capture more detail and fewer to give us statistical power to find those relationships that exist. We have found using simulation studies that three-level discretization works well with our algorithm [9], and used this here.

*iii) Search method*

To find the graph *G* that yields a high score, we used the greedy search heuristic method [7]. Previously, using simulations, we have evaluated three methods from different classes of search techniques—genetic algorithms, simulated annealing, and greedy search—and found that all produced comparable results on networks with a small number of nodes, but that greedy search was the fastest computationally [9]. A greedy search begins with a randomly chosen initial graph. To this graph, addition or deletion of links are evaluated, and the change with the greatest score increase is performed. This process is repeated iteratively until no change improves the score. To escape from local maxima, the search restarts again with a new initial random graph. We used 100 such restarts, and chose the graph with the highest score as the inferred network (Fig. S8). Overall, this process searches approximately 10 million distinct graphs before choosing the inferred network.

*iv) Influence score*

The influence score we developed for DBN algorithms is described in detail elsewhere [9]; the most precise statement of its calculation can be found in the code for the Banjo software which implements it (freely available at [*http://www.cs.duke.edu/~amink/software/*](http://www.cs.duke.edu/~amink/software/)*banjo/*). Briefly, it is a summarization of the conditional probability relationships in a Bayesian network to approximate the sign (excitatory or inhibitory in our case) and magnitude of the interaction between a variable and each of its parents (inputs). This is based on the intuition that if a variable tends to have a high value when its parent is high and a low value when its parent is low, the parent is presumably an activator; conversely, if a variable tends to have a low value when its parent is high and a high value when its parent is low, the parent is presumably a repressor. We calculated the influence score based on the conditional probabilities of the variables in the inferrred network. Because the conditional probabilities, *θ_ijk_* (the probability of the variable *i* taking on state *k* when its parents are in instantiation *j*), are integrated out in the BDe score we need to calculate these values for the inferred network; they are determined from the same *N_ijk_* and *α_ijk_* which already have been calculated for the BDe score as follows:

*θ_ijk_* = .

We then calculate a cumulative distribution function (CDF) *c* of these probabilities for each variable and parent instantiation by summing over all lower states *k'*:

*c_ijk_*=$S_{k^{'}=0}^{k}$*θ_ijk'_*

where *j* and *k* index ordered sets of states. Thus, *c_ijk_* represents the probability that variable *X_i_* (electrode location in our case) is in state *k* or lower when its parents are in instantiation *j*. An activating parent would cause the CDF to shift in the positive direction (right) as the parent’s value increases; vice versa for repressing parents. We pass these *c_ijk_* values through a voting system. Each parent gets one vote for each of the possible configurations of the other parents, e.g., with three parents each having three states, each parent votes nine times for the nine (3*3) possible configurations of the other parents. Voting occurs with the other parents held constant in one of their states, and is based on a comparison of an ordered array of *c_ijk_*’s such that *j-1* indexes the parent state for which the parent in question has a value of one ordered state less than it does in state *j*. If all *c_ijk_*≤ *c_i_*(*_j_*_-1)_*_k_* (CDF shift positive), the vote is for a positive interaction; if all *c_ijk_*≥ *c_i_*(*_j_*_-1)_*_k_* (CDF shift negative), the vote is for a negative interaction; if neither of these hold, the vote is neutral. If all of a parent’s votes are positive or positive and neutral, it is considered an activator and the sign of the influence score is set to be positive (+); if all of a parent’s votes are negative or negative and neutral, it is considered a repressor and the sign of the influence score is set to be negative (–); if the votes are both positive and negative, the sign is indeterminate and the influence score is set to 0. Otherwise, the magnitude of the influence score is equal to the sum of the non-neutral votes of the difference between *c_ij'k_* and *c_ij''k_*, where *j'* and *j''* are the parent instantiations when this parent is at its lowest and highest values, respectively. The score is then divided by the total number of votes (including neutral) to scale it between –1 to 1. The greater the magnitude of the influence score, the more strongly the parent is an activator or repressor (or in our case, the stronger the net positive or negative interaction between brain regions).

*Technical Note*

We note that while the networks we infer are referred to as ‘neural information flow networks’, this terminology does not refer directly to ‘information’ in the information theoretic sense—it is instead a descriptive term used within neuroscience.

**REFERENCES**

1. DiCarlo JJ, Lane JW, Hsiao SS, Johnson KO (1996) Marking microelectrode penetrations with fluorescent dyes. J Neurosci Methods 64: 75-81.

2. Naselaris T, Merchant H, Amirikian B, Georgopoulos AP (2005) Spatial reconstruction of trajectories of an array of recording microelectrodes. J Neurophysiol 93: 2318-2330.

3. Mello CV, Nottebohm F, Clayton D (1995) Repeated exposure to one song leads to a rapid and persistent decline in an immediate early gene's response to that song in zebra finch telencephalon. J Neurosci 15: 6919-6925.

4. Jarvis ED, Nottebohm F (1997) Motor-driven gene expression. Proc Natl Acad Sci U S A 94: 4097-4102.

5. Mello CV, Clayton DF (1994) Song-induced ZENK gene expression in auditory pathways of songbird brain and its relation to the song control system. J Neurosci 14: 6652-6666.

6. Friedman N, Murphy K, Russell S (1998) Learning the structure of dynamic probabilistic networks. Proceedings of the Fourteenth Conference on Uncertainty in Artificial Intelligence. San Francisco, CA: Morgan Kaufmann. pp. 139-147.

7. Heckerman D, Geiger D, Chickering DM (1995) Learning Bayesian networks: The combination of knowledge and statistical data. Mach Learn 20: 197-243.

8. Cowell RG (2001) Conditions under which conditional independence and scoring methods lead to identical selection of Bayesian network models. Proceedings of the 17th Conference in Uncertainty in Artificial Intelligence. San Francisco: Morgan Kaufmann Publishers Inc. pp. 91-97.

9. Yu J, Smith VA, Wang PP, Hartemink AJ, Jarvis ED (2004) Advances to Bayesian network inference for generating causal networks from observational biological data. Bioinformatics 20: 3594-3603.
