# Supplementary material for: Computational Inference of Neural Information Flow Networks
Source: PLoS Comput Biol. 2006 Nov 24;2(11):e161. doi: 10.1371/journal.pcbi.0020161 (PMC1664702; doi:10.1371/journal.pcbi.0020161)
Supplement: Table S1 — (62 KB DOC) [file pcbi.0020161.st001.doc]

Statistics in the table below are organized such that if there is an entry in column *i* and row *j*, it means that the value of the variable for condition *i* is greater than that for condition *j*. The entry indicates how statistically significant this difference is. Since the value for *i* is greater than *j*, it follows that the value for *j* cannot be greater than *i*, and there is no entry in column *j* and row *i.*

Table S1. Statistics for analysis of subsections of stimulus from main text and Figure 3B and C.

| Comparison of number interactions for each subsection (main text)  ANOVA: F_4,20_=0.5, P=0.7, repeated measures  No pair-wise comparisons performed, due to non-significant ANOVA | | | | | |
| --- | --- | --- | --- | --- | --- |
| Comparison of number of links per network for each subsection (Fig. 3B)  ANOVA: F_4,344_=124.5, P<0.0001, repeated measures, controlled for bird | | | | | |
| Bonferonni-corrected pair-wise comparisons: | | | | | |
| stimulus  subsections: | (silence)  before | (sound)  during | (silence)  after | onset | offset |
| before |  | - |  | P<0.001* | P<0.001* |
| during | P=0.013* |  | P>0.5 | P<0.001* | P<0.001* |
| after | P>0.5 | - |  | P<0.001* | P<0.001* |
| onset | - | - | - |  | - |
| offset | - | - | - | P=0.3 |  |
| Comparison of percent of interactions matching interactions from entire stimulus for each subsection (Fig. 3C; main text)  ANOVA: F_4,20_=2.3, P=0.09, repeated measures  No pair-wise comparisons performed, due to non-significant ANOVA | | | | | |
| Comparison of percent of links matching interactions from entire stimulus for each subsection (main text)  ANOVA: F_4,304_=2.0, P=0.09, repeated measures, controlled for bird  No pair-wise comparisons performed, due to non-significant ANOVA | | | | | |

Asterisks (*) indicate significant differences. A P-value in a cell indicates that the percent of links for the stimulus subsection for that column is greater than for that row. For example, the number of links for ‘onset’ subsection is greater than the number for ‘before’ subsection at P<0.001.
