# Supplementary material for: Computational Inference of Neural Information Flow Networks
Source: PLoS Comput Biol. 2006 Nov 24;2(11):e161. doi: 10.1371/journal.pcbi.0020161 (PMC1664702; doi:10.1371/journal.pcbi.0020161)
Supplement: Table S2 — (51 KB DOC) [file pcbi.0020161.st002.doc]

Statistics in the table below are organized such that if there is an entry in column *i* and row *j*, it means that the value of the variable for condition *i* is greater than that for condition *j*. The entry indicates how statistically significant this difference is. Since the value for *i* is greater than *j*, it follows that the value for *j* cannot be greater than *i*, and there is no entry in column *j* and row *i.*

Table S2. Statistics for comparison of proportion of links of each length of Figure S4.

| ANOVA: F_5,430_=58.8, P<0.0001, repeated measures, controlled for bird | | | | | | |
| --- | --- | --- | --- | --- | --- | --- |
| Bonferonni-corrected pair-wise comparisons: | | | | | | |
| proportion of links: | length 1 | length 2 | length 3 | length 4 | length 5 | length 6 |
| length 1 |  | - | - | - | - | - |
| length 2 | P<0.0001* |  | - | - | - | - |
| length 3 | P<0.0001* | P<0.0001* |  | P=0.003* | P=1.0 | - |
| length 4 | P<0.0001* | P=0.036* | - |  | - | - |
| length 5 | P<0.0001* | P<0.0001* | - | P=0.1 |  | - |
| length 6 | P<0.0001* | P<0.0001* | P=1.0 | P=0.02* | P=1.0 |  |

Asterisks (*) indicate significant differences. A P-value in a cell indicates that the proportion of links of the length for that column is greater than for that row. For example, proportion of length 2 is significantly more frequent than length 4 at P=0.036. Grey shaded cells indicate comparisons that cannot be done, a condition against itself.
