# Supplementary material for: Computational Inference of Neural Information Flow Networks
Source: PLoS Comput Biol. 2006 Nov 24;2(11):e161. doi: 10.1371/journal.pcbi.0020161 (PMC1664702; doi:10.1371/journal.pcbi.0020161)
Supplement: Table S3 — (57 KB DOC) [file pcbi.0020161.st003.doc]

Statistics in the table below are organized such that if there is an entry in column *i* and row *j*, it means that the value of the variable for condition *i* is greater than that for condition *j*. The entry indicates how statistically significant this difference is. Since the value for *i* is greater than *j*, it follows that the value for *j* cannot be greater than *i*, and there is no entry in column *j* and row *i.*

Table S3. Statistics for edit distance comparisons in Figure 5A-C.

| Comparison of networks from noise stimuli (plain and amplitude-modulated noise) and song stimuli (Fig. 5A)  ANOVA: F_2,648_=29.1, P<0.0001, controlled for bird | | | |
| --- | --- | --- | --- |
| Bonferroni-corrected pair-wise comparisons: | | | |
| edit distances: | within noise | within song | across noise and song |
| within noise |  | P=0.0003* | P<0.0001* |
| within song | - |  | P=0.01* |
| across noise and song | - | - |  |
| Comparison of networks from plain noise and amplitude-modulated noise (Fig. 5B)  ANOVA: F_2,137_=3.3, P=0.04, controlled for bird | | | |
| Bonferroni-corrected pair-wise comparisons: | | | |
| edit distances: | within plain noise | within modulated noise | across plain and modulated noise |
| within plain noise |  | P=0.03* | P=0.5 |
| within modulated noise | - |  | - |
| across plain and modulated noise | - | P=0.07 |  |
| Comparison of networks from two sets of songs (Fig. 5C)  ANOVA: F_2,137_=0.7 P=0.5, controlled for bird  No pair-wise comparisons performed, due to non-significant ANOVA | | | |

Asterisks (*) indicate significant differences. A P-value in a cell indicates that the edit distance of the group for that column is greater than for that row. For example, edit distance ‘within song’ networks is greater than edit distance ‘within noise’ networks at Bonferroni-corrected P=0.0003.
