# Supplementary material for: Computational Inference of Neural Information Flow Networks
Source: PLoS Comput Biol. 2006 Nov 24;2(11):e161. doi: 10.1371/journal.pcbi.0020161 (PMC1664702; doi:10.1371/journal.pcbi.0020161)
Supplement: Video S1 — (47 KB PPT) [file pcbi.0020161.sv001.ppt]

## Slide 1
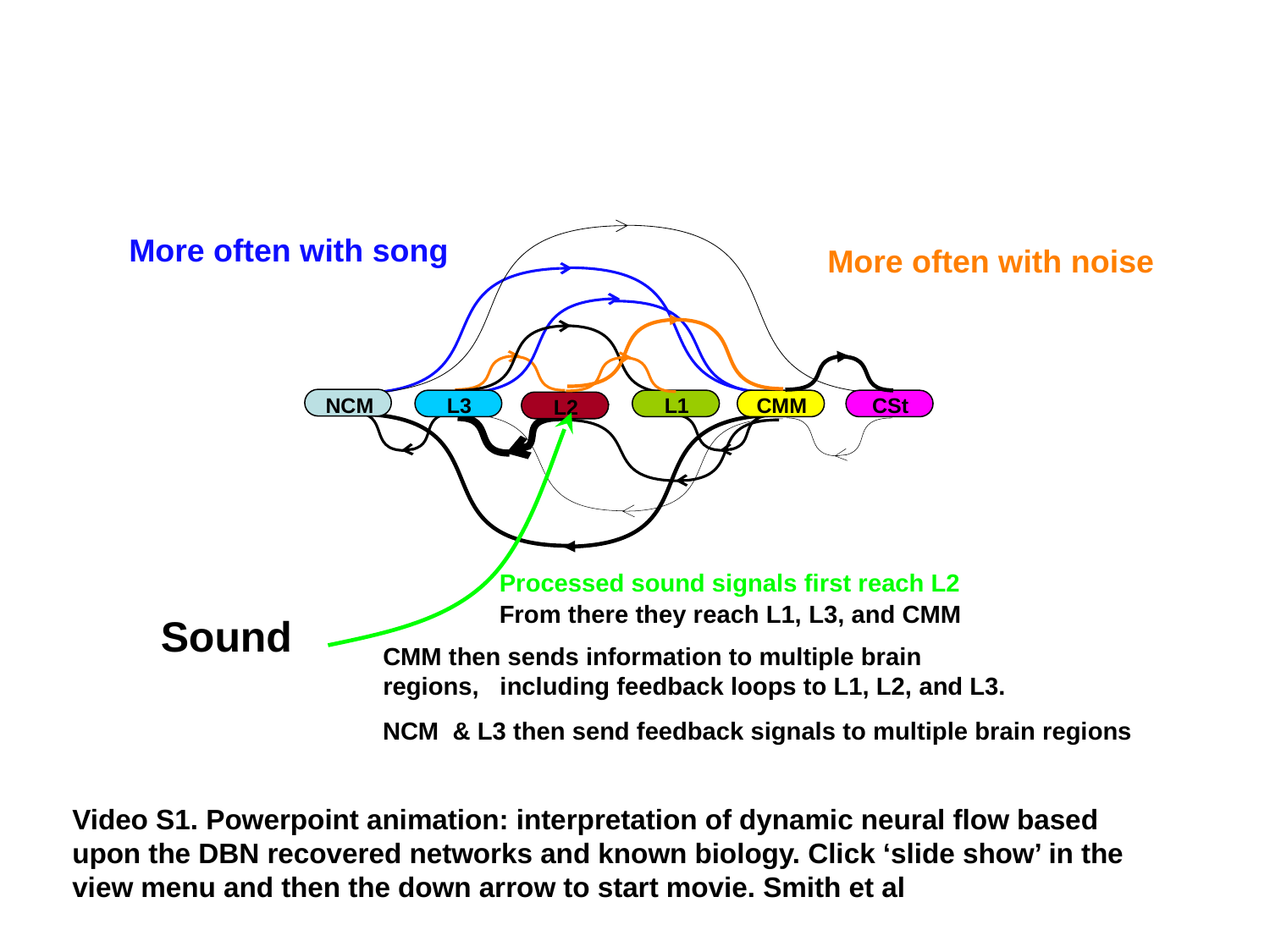

More often with song
More often with noise
NCM
L3
L1
CMM
CSt
L2
Processed sound signals first reach L2
From there they reach L1, L3, and CMM
Sound
CMM then sends information to multiple brain regions, including feedback loops to L1, L2, and L3.
NCM & L3 then send feedback signals to multiple brain regions
Video S1. Powerpoint animation: interpretation of dynamic neural flow based upon the DBN recovered networks and known biology. Click ‘slide show’ in the view menu and then the down arrow to start movie. Smith et al
